# Spatial genetic structure and diversity of natural populations of *Aesculus hippocastanum* L. in Greece

**DOI:** 10.1371/journal.pone.0226225

**Published:** 2019-12-11

**Authors:** Łukasz Walas, Petros Ganatsas, Grzegorz Iszkuło, Peter A. Thomas, Monika Dering

**Affiliations:** 1 Institute of Dendrology, Polish Academy of Sciences, Parkowa, Kórnik, Poland; 2 Aristotle University of Thessaloniki, School of Forestry and Natural Environment, Laboratory of Silviculture, Thessaloniki, Greece; 3 Faculty of Biological Sciences, University of Zielona Góra, Prof. Z. Szafrana, Zielona Góra, Poland; 4 School of Biological Sciences, Keele University, Staffordshire, United Kingdom; 5 Harvard Forest, Harvard University, Petersham, MA, United States of America; 6 Faculty of Forestry, Poznań University of Life Sciences, Wojska Polskiego, Poznań, Poland; Chinese Academy of Sciences, CHINA

## Abstract

Horse-chestnut (*Aesculus hippocastanum* L.) is an endemic and relict species from the Mediterranean *biodiversity hotspot* and a popular ornamental tree. Knowledge about the evolutionary history of this species remains scarce. Here, we ask what historical and ecological factors shaped the pattern of genetic diversity and differentiation of this species. We genotyped 717 individuals from nine natural populations using microsatellite markers. The influence of distance, topography and habitat variables on spatial genetic structure was tested within the approaches of isolation-by-distance and isolation-by-ecology. Species niche modeling was used to project the species theoretical range through time and space. The species showed high genetic diversity and moderate differentiation for which topography, progressive range contraction through the species’ history and long-term persistence in stable climatic refugia are likely responsible. A strong geographic component was revealed among five genetic clusters that are connected with very limited gene flow. The environmental variables were a significant factor in the spatial genetic structure. Modeling results indicated that future reduction of the species range may affect its survival. The possible impact of climate changes and high need of *in situ* conservation are discussed.

## Introduction

During the Cenozoic (66–23.03 Ma) period, Arcto-Tertiary flora covered a large part of the Northern Hemisphere and formed a unique type of forest ecosystem [1–3]. Species of Tertiary humid-temperate forests began to decline rapidly along with the aridization that appeared in the middle Miocene and were finally pushed out during cold periods of the Pleistocene into warm and humid refugia located in the Central Asia and the West Coast of the North America [4]. Many Tertiary species found safe shelter in the Mediterranean Basin, which is one of the important refugia for species of this ancient flora [5].

Accumulation of endemic taxa drives the formation of biodiversity hotspots and most conservation effort is expended on these endemic species due to their high extinction risk [6, 7]. The Mediterranean Basin is one of the biodiversity hotspots with an exceptional role in the preservation of unique species and genetic diversity [6, 8]. Currently, climate change is becoming a serious and the most urgent problem in this region. A wide spectrum of biological and socio-economic consequences are predicted that impose a challenge for the conservation of the biodiversity stored in the Mediterranean biodiversity hotspot [9–11]. Expected environmental changes may cause extinction of many endemic species which can persist only in a stable climate refugia [12]. Therefore, the protection of the Mediterranean relicts and endemics is an extremely urgent issue and produces a number of challenges.

Firstly, the persistence of Tertiary relicts depends on the conservation of the habitats to which they are frequently highly specialized [13, 14]. In the densely populated Mediterranean, increasing demand on land for agriculture and urban development means that protecting habitats for Tertiary relict and other narrowly distributed plants is difficult [15, 16]. Secondly, long-term survival of relict and endemic species depends on the genetic diversity which is a measure of the potential within a population to respond to natural selection [17, 18]. There are many studies concerning genetic diversity patterns and their causal factors in plants, especially trees, which are key elements of forest ecosystems and in the forest industry [19]. Less attention is generally paid to rare tree species with low economic importance. However, some of these can have high biodiversity impact. Study of relict and endemic species allows better understanding of the processes of extinction and survival, which is especially relevant in the context of expected climate-induced decline in worldwide populations [20–24].

Two basic sets of factors drive population genetic structure and divergence. One of them is the historical biogeography including historical climatic change and tectonic movements that have modified genetic connectivity among populations resulting in significant isolation-by-distance (IBD) [25, 26]. In the Mediterranean region, geological history has left strong and still readable signatures in the current spatial organization of genetic diversity and differentiation [27–30]. The second set of factors affecting the genetic structure are environmental variables including edaphic conditions and topographic heterogeneity that may drive adaptive divergence [31]. Habitat conditions are especially important to endemic and relict species as their populations tend to occur in a very narrow range of environmental conditions [13, 32–34]. Populations existing in isolated areas with dissimilar conditions, such as refugia, may undergo divergent selection that can lead to local adaptation and genetic divergence detectable at neutral variability [35]. This isolation-by-ecology (IBE), referring to significant negative correlation between genetic distance and ecological dissimilarity (edaphic conditions and topography), has recently become recognized as an almost equally important driver of intra-specific differentiation and speciation as the classic IBD model [31, 36, 37].

Horse-chestnut (*Aesculus hippocastanum* L.) is a Tertiary relict and naturally occurs only in the Balkan Peninsula [38]. During the Pliocene, *Aesculus* was broadly distributed in Europe and paleaobotanical records prove its presence in Africa and the Caucasus [39, 40]. Climate change in the Pleistocene pushed the species into mountain refugia in the Balkan Peninsula. The natural origin of horse-chestnut remained unknown until the end of 19^th^ century, undoubtedly because of its dispersed and low-density occurrence in the high mountains [38, 41–43]. Paradoxically, the species has been widely cultivated as an ornamental tree in Europe since the 16^th^ century [41].

As a paleoendemic of Tertiary origin, horse-chestnut has experienced a long and rich history during which it has experienced several major geological events that were crucial in the development of spatial genetic structure of tree species in the Mediterranean region [44]. Hence, we postulate that the historical factors might be major determinants of the species’ genetic diversity and differentiation patterns. Additionally, being an endemic species, horse-chestnut may show reduced genetic diversity [45, 46] which would be detrimental to retaining evolutionary potential [47, 48]. Accordingly, the International Union for Conservation of Nature has recommended urgent genetic research on natural stands of horse-chestnut [49].

In its favour, horse-chestnut is distributed across a large mountain system stretching *c*. 425 km along the Balkan Peninsula, implying the possibility of abrupt environmental gradients. Similar to other Tertiary relicts, the species requires humid conditions [13, 50, 51], and shows some habitat specialization [52]. According to Tsiroukis [53], 62% of natural populations are located in ravines with constantly flowing water and 11% with temporary water. The remaining small fraction is present in dry ravines or on arid and steep slopes (9% and 6%, respectively). Hence, apart from the historical factors, dissimilar ecological conditions might also potentially promote genetic differentiation in horse-chestnut through adaptive divergence, which relates to IBE [54, 55].

In this study we examined genetic diversity and differentiation within natural populations of horse-chestnut in Greece. We integrated genetic methods with ecological niche modeling to better understand the processes and factors that have governed species evolution since the Pleistocene. Such analyses could help to disentangle the roles of historical and ecological factors in shaping the species’ spatial genetic structure. We hypothesized that: (1) spatial genetic structure of horse-chestnut is mostly determined by historical factors, and the species natural populations are characterized by low genetic variability within and high genetic diversity among populations as expected for a palaeoendemic; (2) the range of horse-chestnut will be reduced in the future due to climate change.

## Material and methods

### Species description and population sampling

Horse-chestnut is native to mountainous regions of the Balkan Peninsula, particularly Greece, where it occurs at altitudes between 228 m and 1485 m a.s.l. [38]. The species is andromonoecious with the majority of flowers functionally male (c. 73%) [56]. Flowers, which are pollinated by insects, are an important source of nectar [57, 58]. However, horse-chestnut is ambophilous since some pollen can also be spread by wind [59]. Large, heavy seeds are dispersed mostly by gravity [60]. They may be secondarily dispersed by water since natural populations occur mostly along streams [53]. Seeds of the related Japanese horse-chestnut (*Aesculus turbinata* Blume) travel up to 14.5 m from the parent tree with a mean distance 12.2–44.7 m [61]. These distances are probably similar in horse-chestnut.

Due to fragmentary information of species distribution in Balkans, collection of the material in this study was limited to the core range of the species in Greece. Sites with the highest number of mature individuals were selected for investigation using data from Avtzis et al. [38]. Since most of the stands were characterized by small populations [38], we included nine of these in our study to ensure reliable statistical estimations based on microsatellite markers. Material was collected in June 2015. All field sampling was organized by the Laboratory of Silviculture of the Aristotle University of Thessaloniki, and was carried out in close collaboration with the local Forest Services. More specifically, field sampling was carried out after communication with the Directors of the local Forest Districts, and always arranging a local staff accompanies the research team for sampling procedure. Horse-chestnut is not a protected species in Greece and materials were not collected in protected areas, therefore special permissions were not required. In total, 717 mature individuals were sampled (Table 1, S1 Table). Additionally, to analyze how genetic diversity is transferred from adult trees to offspring, 191 one-year-old seedlings were collected in three populations that showed good regeneration (Karitsa I, Karitsa II and Perivoli). All sampled populations are distributed in three mountain ranges: Pindos Mts. (Ondria, Kalampaka, Dasos Nanitsa, Vaeni, Vathirrevma, Perivoli), Ossa Massif (Karitsa I and Karitsa II) and the more southerly located Parnassus Massif (Mariolata). Most of analyzed populations grew in the vicinity of streams; the exceptions were populations Ondria and Mariolata.

**Table 1:** Location and clonal structure of the studied populations.

Population	Voucher	Latitude	Longitude	Altitude [m a.s.l.]	Number of individuals	Number of genotypes	R
**Dasos Nanitsa**	KOR 51216	39°42' N	21°21' E	1029	84	83	0.99
**Kalampaka**	No voucher	39°48' N	21°16' E	1371	23	23	1.00
**Mariolata**	KOR 51230 KOR 51219	38°37' N	22°26' E	1239	46	46	1.00
**Ondria**	KOR 51217 KOR 51218	40°20' N	21°05' E	1463	48	48	1.00
**Vaeni**	No voucher	39°12' N	21°42' E	1089	33	29	0.88
**Vathirrevma**	No voucher	39°25' N	21°25' E	1028	78	75	0.96
**Karitsa I—seedlings**	KOR 51280	39°48' N	22°45' E	705	50	50	1.00
**Karitsa I—mature**	KOR 51280	39°48' N	22°45' E	705	114	94	0.82
**Karitsa II—seedlings**	No voucher	39°50' N	22°42' E	950	46	46	1.00
**Karitsa II—mature**	No voucher	39°50' N	22°42' E	950	22	22	1.00
**Perivoli—seedlings**	KOR 51226	39°58' N	21°11' E	915	95	95	1.00
**Perivoli—mature**	KOR 51226	39°58' N	21°11' E	915	78	75	0.96

R–level of clonality

We noticed root suckers of *Aesculus* during the field study, but a full determination of the connections between individuals was not possible. We used a random pattern of sampling but estimated the level of clonality in each population using a clonal diversity parameter (genotypic richness) *R = (G—1) / (N—1)*, in which *G* denotes the number of distinct genotypes scored in a population (genets) and *N* is the total number of trees sampled (ramets) [62]. The values of *R* range from 1, where each genotype is unique, to 0, when all sampled individuals have the same genotype (Table 1).

### DNA isolation and genotyping

Genomic DNA was extracted from leaf tissue using protocols described by Dumolin et al. [63]. To determine the genetic variation and genetic diversity, 10 nuclear microsatellite loci (nSSRs) developed for *A*. *turbinata* were tested [64]. Preliminary analysis allowed final selection of eight polymorphic loci: AT3D6, AT5D2, AT6D2, AT6D8, AT6D11, AT6D12, AT7D1 and AT7D8. Two rejected primer pairs (AT6D17 and AT5D10) gave low quality products. PCRs were conducted in a final volume of 10 μL, containing 1 × reaction buffer with 2.5 mM MgCl_2_, 2 μM of dNTP mix, 0.5 U of VivaTaq polymerase (Novazym, Poznań, Poland), 0.4 μM of each starters and 100 ng of DNA. Reactions were conducted using the following thermal protocol: initial denaturation at 94°C/12 min., followed by 35 cycles of denaturation at 94°C/30 s, annealing at 52°C or 55°C (specific to locus) for 30 s, elongation at 72°C/60 s and final elongation at 72°C for 5 min. Products of amplification were analyzed using a 3130 Genetic Analyzer (Applied Biosystems, Foster City, California, USA) with internal size standard GeneScan LIZ-500 and genotypes were scored using GENEMAPPER vs. 4.0 (Applied Biosystems, Foster City, California, USA).

### Genetic diversity and differentiation

All clones were removed before analysis of genetic data, identified using the “Find Clones” procedure in GENEALEX 6.4 [65]. This software was also used to estimate basic multilocus within-population diversity estimates such as mean number of alleles (*A*), effective number of alleles (*A*_*e*_) and number of private alleles (*A*_*p*_). INEST v. 2.0 [66] was used to calculate observed (*H*_*o*_) and expected (*H*_*e*_) heterozygosity while FSTAT v 2.9.3. [67] was used to compute allelic richness (*A*_*r*_). A Bayesian approach implemented in INEST software was applied to estimate the inbreeding coefficient (*F*_*IS*_) including a ‘null alleles’ correction according to the individual inbreeding model (*IIM*). The estimation was run with 500,000 MCMC cycles with every 200^th^ updated and a burn-in of 50,000. The Deviance Information Criterion (*DIC*) was used to compare the full model (‘nfb’, when F_*IS*_>0) with the random mating model (‘nb’, when *F*_*IS*_ = 0) to assess the determinants of levels of homozygosity. The significance of the heterozygote deficiency in a sampled population was tested by U test [68] in GENEPOP [69], and *p*-values were obtained with the Markov chain algorithm using default settings. Frequency of the null alleles (*Null*) and Wright’s fixation index (*F*_*ST*_) were estimated in FREENA with ENA (Excluding Null Alleles) correction [70]. This method was used to correct positive bias inducted by the presence of the null alleles. The hypothesis on the bottleneck effect in the demographic history of the horse-chestnut populations was tested with the *M*-ratio approach implemented in INEST.

Subdivision of the horse-chestnut gene pool was evaluated by a non-spatial Bayesian clustering model implemented in STRUCTURE 2.3.4. [71]. The procedure for each of the 10 independent runs included 10^4^ of burn-in and 10^5^ MCMC iterations with the maximum number of clusters set to K = 10. The STRUCTURE model assumed correlated allele frequencies within populations and allowed for mixed ancestry of individuals. To estimate the best-supported number of clusters (S1 Fig; see Supplemental Data with this article), Evanno's delta K method implemented in CLUMPAK [72] was used. Individuals that had an assignment for each cluster of less than 70% were recognized as admixed. In order to detect hidden population-substructure, additional STRUCTURE analysis for separate populations with K = 5 was performed with the same initial conditions. INSTRUCT software [73] was used to verify STRUCTURE results and to estimate selfing rate in each genetic cluster. Analyze was performed for K = 5 as two independent chains in mode 2 (infer populations structure and selfing rates) with Adaptive Independence Sampler, 10^6^ MCMC iterations and 5×10^5^ of burn-in.

In addition to the model-based approach, a discriminant analysis of principal components (DAPC) [74] was performed to infer homogenous genetic clusters. The main advantage of this method is that it works in the absence of any assumptions related to population genetic models and it is a quick computation. DAPC was conducted in the package ‘adegenet’ in R 3.4.3 [75, 76]. In contrast to STRUCTURE, this analytical method can detect hierarchical patterns in the spatial genetic structure and does not require *a priori* group definition [74]. DAPC is a multivariate two-stage procedure in which data are firstly transformed by principal component analysis (PCA) to remove correlation between variables, which are then submitted to discriminant analysis (DA). This part of the procedure aims at partitioning the total genetic variability into between-cluster and within-cluster components. The procedure optimizes the variance for the first component and minimizes the within-cluster component to obtain the best discriminative power and to define final clusters. To infer the optimal number of the genetic clusters function ‘*find*.*cluster’* was used, which runs successive K-means clustering with increasing number of clusters *K* from 2 to 50 supported by Bayesian Information Criterion (BIC). Function ‘*xvalDapc*’ with 30 replications was used to perform cross-validation on varying numbers of principle components. DAPC was conducted with the function ‘*dapc*’. Fourteen PCs and an optimal number of clusters (10) were applied in estimations. Output was visualized using library ‘ggplot2’ in R [77]. The program POPULATIONS 1.2.30 was used to construct a phylogenetic tree, which shows the relationships among all genetic clusters determined by STRUCTURE [78]. A Cavalli-Sforza and Edwards (*D*_*c*_) genetic distances was used to obtain a neighbor-joining (NJ) tree.

BAYESASS 3.0 (BA3) was used to infer recent migration rates among studied populations [79]. BA3 is based on the Bayesian MCMC method and estimates migration over the last few generations without assumptions on the Hardy-Weinberg equilibrium. It works best when migration is relatively low [80], which was expected in these isolated populations of horse-chestnut. To obtain a recommended acceptance rate within the range of 20–40% [81], MCMC mixing parameters were initially adjusted during the testing stage for inbreeding coefficient (0.30) and migration rate (0.75) which resulted in acceptance rates of 23% and 28%, respectively. Default settings of mixing parameters for allele frequencies were used. Afterwards, the final analysis was conducted with 10^7^ MCMC iterations, 10^6^ of burn-in and sapling every 1000^th^ iteration. To check consistency, three independent runs with different random seeds were carried out. Finally, a script of Meirmans [80] applied in R [76] was used to calculate the Bayesian deviance for each run. The run with the lowest value of the Bayesian deviance was chosen and used for our interpretations.

Historical gene flow among populations was investigated with MIGRATE-n v. 3.2.6 [82, 83]. This tool computes mutation-scaled historical effective population size (*Θ*) and migration rates (*M*) using a coalescence approach. All runs used the Bayesian inference method and the Brownian motion mutation model with identical priors and parameter values, and identical mutation rates were assumed among loci. Testing runs were conducted to set uniform priors (minimum, maximum, delta) for *Θ* and *M* (0; 10000; 1000) for all subsequent runs. Each run finally consisted of 50,000 recorded steps at an increment of 100 steps, after a burn-in of 20,000 steps, and a static heating scheme (four chains set at 1, 1.5, 3, 10^5^). We performed three independent runs with different initial seed numbers to verify consistency and used the Bezier approximation for the marginal likelihood to test which run best fit the data [83].

### Climate niche modeling

The program MAXENT version 3.3.2 [84, 85] was used to build a climate niche model. This software uses a maximum entropy presence-only model to estimate probability of species occurrence. The dataset of 74 known localities (S1 Table) was collected from the available literature on horse-chestnut distribution [38, 42, 86–88]; each population was represented by a single point analyses. Bioclimatic variables with a resolution of 30 arc-sec were downloaded from the WorldClim database [89] but seven out of 19 variables were excluded because of strong correlation (S2 Table) evaluated using ENMTOOLS v1.3. [90]. Variables for the Mediterranean Basin and Europe were used. Analyses were performed for the present day as well as for two past periods (maximum glaciation *c*. 22 000 years ago and mid-Holocene *c*. 6 000 years ago) and for future conditions. Three scenarios of climate changes were used for the future projection: RCP 2.6 (Representative Concentration Pathway)—increase of average temperature by 1°C before the year 2065; RCP 4.5: + 1.4°C before the year 2065; and RCP 8.5: + 2°C before the year 2065 [91]. The Community Climate System Model (CCSM) of global climate was used [92]. Analyses were performed as a bootstrap with 100 replicates; in each replication 20% of the data were set aside as test points and a ‘random seed’ option was applied. The maximum iterations were set to 10,000, convergence threshold to 0.00001 and output was set to logistic. A Receiver Operating Characteristic (ROC) curve and Value of Area Under the Curve (AUC) were used to evaluate the model [93, 94]. Models included areas of Europe, North Africa and the Middle East. Results of analyses were visualized in QGIS 2.18.20 ‘Las Palmas’ [95].

### Isolation-by-ecology and isolation-by-distance

A connectivity-resistance approach implemented in the software CIRCUITSCAPE v.4.0.5 [96] was applied to test the significance of the three possibly important factors in gene flow among horse-chestnut populations (distance, topographic complexity and habitat suitability) within the approach of IBE. CIRCUITSCAPE is based on the electrical circuit theory. It models the gene flow (connectivity) between pairs of populations as the resistance distance between nodes connected by resistors. The resistance surfaces are created by different landscape properties that may impede or modify the gene flow and the landscape itself is considered as an electric network. The genetic distance that is built between populations, and is attributable to landscape resistance, is termed isolation-by-resistance (IBR). The unique property of the CIRCUITSCAPE is that each added additional pathway and connection between nodes reduces the resistance. In other words, the conductance of the resistors reflects the intensity of gene flow between populations [97].

Three different resistance surfaces were developed as matrices. A matrix for isolation-by-distance model (IBD) was created using completely flat landscapes in the program QGIS 2.14.21 [95]; this software was also used for creating a relief raster to topographic complexity calculations. To test if the past or present environmental conditions were relevant for observed differentiation pattern (*i*.*e*. IBE), past and present layers of the niche suitability previously obtained with MAXENT were used [84, 85]. Matrices of habitat suitability were used as conductance surfaces. All matrices were tested against the matrices of the genetic distance (pairwise *F*_*ST*_ and *F*_*ST*_*NA* values) with a multiple matrix regression approach implemented in R (function *‘mantel*.*rtest’*).

## Results

### Genetic diversity

Out of 717 individuals genotyped, 686 unique genotypes were identified (Table 1). In most populations distinct genotypes were detected (*R = 1*) or only a few individuals (1–3) were of vegetative origin (Table 1). The highest level of clonality (*R =* 0.82) was noted in Karitsa I, where 94 unique genotypes were detected among 114 trees sampled.

Screening the sampled individuals with eight nSSR gave 124 different alleles in total and number of alleles varied from 48 in Kalampaka to 88 in Karitsa I—mature (S3 Table). The number of alleles per locus varied from 1–3 (AT6D11) to 8–18 (AT6D12) and was similar to values for *Aesculus turbinata* [64]. The highest value of expected heterozygosity (*H*_*e*_) was at locus AT7D1 in population Dasos Nanitsa (0.915) while the lowest (0.000) at AT6D11 in populations Kalampaka and Ondria, because this locus was monomorphic in these populations. The highest frequency of null alleles was observed in the AT6D2 locus in population Perivoli–mature (23.7%). The effective number of alleles was high at locus AT7D1 in Dasos Nanitsa and Vathirrevma (10.77 and 10.03, respectively), while in the Karitsa I population the highest values were at the AT6D12 (11.50) and AT7D8 (10.44) loci.

Estimators of the genetic diversity are presented in Table 2. The average number of alleles in a population (*A*) ranged from 6.00 in Kalampaka to 11.00 in Karitsa I. Average effective number of alleles (*A*_*e*_) is 4.19, which is much smaller value than average number of alleles (8.73). The highest value of allelic richness (*A*_*r*_) was observed in Vathirrevma (8.44), whereas the lowest was in Kalampaka (5.93). Observed heterozygosity (*H*_*o*_) ranged from 0.442 (Mariolata) to 0.636 (Vathirrevma), while gene diversity (*H*_*e*_) varied from 0.533 (Mariolata) to 0.746 (Vathirrevma). Significant positive *F*_*IS*_ values, suggesting an excess of homozygotes, were observed for all analyzed populations. In Kalampaka, the inbreeding coefficient was highest, reaching 0.1520. According to DIC criterion, in the majority of the populations inbreeding was the likely reason for excess homozygotes. In three populations (Karitsa II–mature, Perivoli–mature and Perivoli–seedlings), null alleles are probably responsible for increased homozygosity level. Generally, null alleles were present in all populations with an average frequency of 6.3%. Private alleles were detected in almost all studied stands, and the highest number (4) was noted for Ondria, located at the northernmost part of the Pindos Mts. (Table 2).

**Table 2:** Parameters of genetic diversity of the studied populations.

Population	N	A	A_e_	A_r_	A_p_	Null	H_o_	H_e_	F_IS_
**Dasos Nanitsa**	83	9.63	4.68	7.22	1	0.052	0.590	0.670	0.0305***
**Kalampaka**	23	6.00	3.82	5.93	0	0.096	0.449	0.644	0.1520***
**Mariolata**	46	7.00	2.77	5.95	0	0.063	0.442	0.533	0.0742***
**Ondria**	48	9.00	4.48	7.73	4	0.053	0.536	0.623	0.0510 ***
**Vaeni**	29	8.63	4.97	8.10	3	0.060	0.573	0.705	0.1093***
**Vathirrevma**	75	10.75	5.34	8.44	2	0.068	0.636	0.746	0.0435***
**Karitsa I–mature**	94	11.00	5.64	8.35	1	0.050	0.593	0.677	0.0410******
**Karitsa I—seedlings**	50	9.25	4.37	7.61	0	0.070	0.543	0.664	0.1014***
**Karitsa II—mature**	22	6.63	3.69	6.56	0	0.041	0.536	0.605	0.0238null***
**Karitsa II—seedlings**	46	8.13	3.72	6.66	1	0.050	0.521	0.603	0.1016***
**Perivoli—mature**	75	9.63	3.96	7.22	1	0.081	0.593	0.711	0.0144null***
**Perivoli—seedlings**	95	9.13	2.83	6.48	0	0.069	0.504	0.607	0.0200null***
**Average**		**8.73**	**4.19**	**7.19**	**1.1**	**0.063**	**0.543**	**0.649**	

N–number of samples, A–average number of alleles, A_e_−effective number of alleles, A_r_−allelic richness, Ap−number of private alleles, Null–frequency of null alleles, H_o_−observed heterozygosity, H_e_−expected heterozygosity, F_IS_−fixation index.

***—departure from HWE at P<0.001.

null***—the random mating model was more probable than the full model.

Genetic diversity between mature parental populations and 1-year-old seedlings was very similar as indicated by *H*_*e*_ values, with the exception of *A*_*r*_ that appeared lower in seedlings (Table 2). However, allelic richness differences between mature and seedlings populations was statistically insignificant (Kruskall-Wallis test, P = 0.513). The allele frequency spectra showed that some alleles were absent in progeny. The average frequency of lost alleles in seedlings from Karitsa I was 4.48%, in Karitsa II 2.88%, and in Perivoli 2.89%. New alleles were also detected in all seedling populations. In Perivoli and Karitsa I the gain of new alleles was low (1.61% and 1.01%, respectively) but a surprisingly high percentage of new alleles, 6.08%, was noted in Karitsa II.

### Spatial genetic structure

CIRCUITSCAPE indicated significant IBD (r = 0.49, P *=* 0.0034) for the set of analyzed populations. Statistically significant global *F*_*ST*_ values with and without the ENA correction attained similarly moderate values (*F*_*ST*_ = 0.109, *F*_*ST*_ = 0.114, respectively), but the pairwise estimations showed a wider range of variation (S4 Table). The highest value of *F*_*ST*_ with ENA correction was noted between Mariolata and Karitsa II (0.227), while the lowest was between the two closest stands, Karitsa I and Karitsa II (0.035). M-ratio test indicated a significant bottleneck effect in all studied populations except for Dasos Nanitsa (P *=* 0.125).

Analysis of recent migration rates among nine studied populations with BAYESASS showed that migration is very low (S2 Fig, S5 Table). The average proportion of migrants was 0.011, indicating that only 1.1% of individuals in a population are migrants. Higher migration was, however, detected between Karitsa I and Karitsa II (0.060) and between Vaeni and Kalampaka (0.042). Historical migration, estimated with MIGRATE-n, was more intensive (S3 Fig, S6 Table). Average numbers of migrants per generation (*θM* / 4) was 133.74. The highest migration was noted from Karitsa I to Karitsa II (1202.21), which can be readily explained by the short distance between these stands. Generally, the gene flow was asymmetrical and more intensive from west (Pindos) to east (Ossa) than in the opposite direction (S3 Fig, S6 Table). The exception to this generality may be the intensive gene flow from Karitsa I to Kalampaka (300.89). The lowest effective population size was in Vaeni (*θ* = 13.56) and the highest in Karitsa II (*θ* = 41.65).

**Fig 1:**
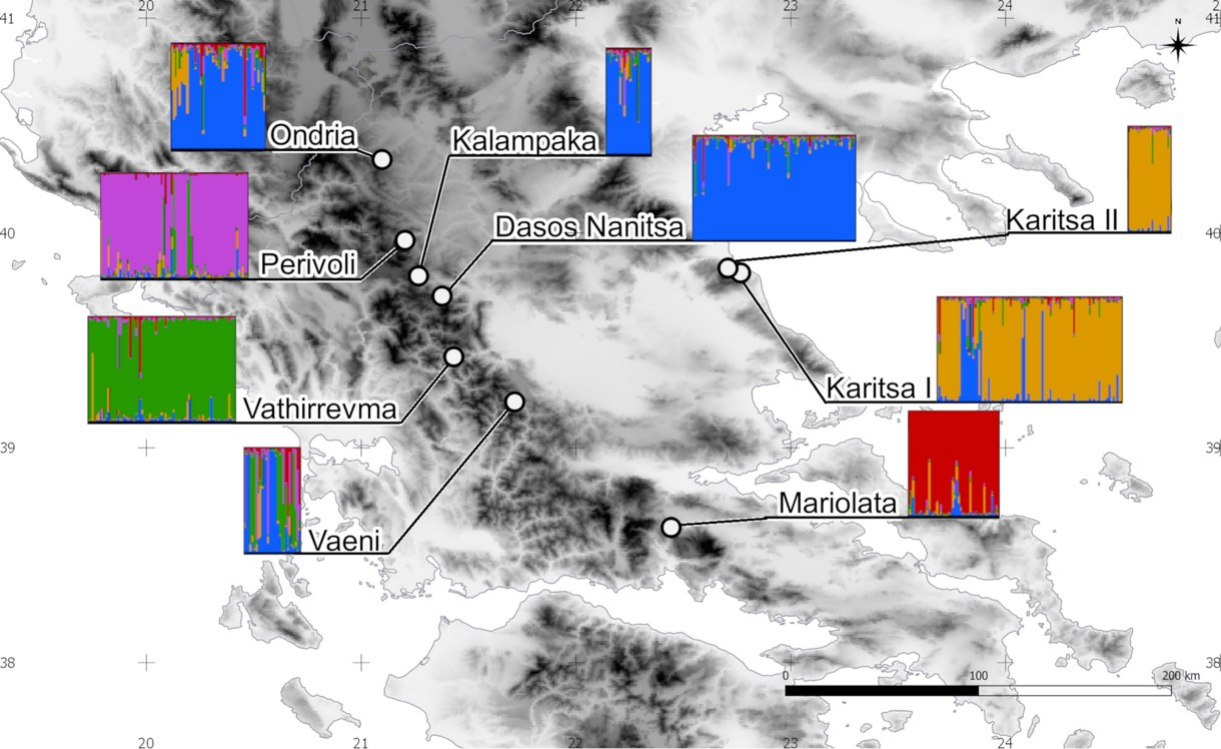
Location of the sampled populations with results of clustering analyses made with STRUCTURE.

The Bayesian analysis of spatial genetic structure made with STRUCTURE defined five homogenic genetic clusters among nine analyzed mature populations (Fig 1, S1 Fig, S7 Table). Three of the clusters consisted of a single population (Perivoli–cluster I, Vathirrevma–cluster II, Mariolata–cluster III), while cluster IV grouped populations from Karitsa I and Karitsa II. Cluster V was formed by the populations of Ondria, Kalampaka and Dasos Nanitsa (Fig 1). The Vaeni population exhibited a wide intermixing within all detected clusters but had a slightly higher percentage of membership to cluster V (34.81%) than to cluster II (30.12%). An interesting result is the presence of a significant genetic admixture in Karitsa I located in the Ossa Massif. Phylogenetic analysis made for five detected genetic clusters showed a division into east populations (Mariolata and Ossa Massif, clusters III and IV) and west populations (three clusters from Pindos Mts.: clusters I, II and V). The cluster from Perivoli turned out to be more similar to the south Pindos cluster than to north Pindos (S4 Fig). Eight out of nine analyzed mature populations showed a division into subpopulations (S5A Fig). In Mariolata two subpopulations were detected, in Vaeni three, in Dasos Nanitsa four; for Karitsa I, Vathirrevma and Perivoli the best K was 5. (S6 Fig). Only in Karitsa I was a substructure not detected (S5A Fig). STRUCTURE analyze for three seedling populations divided young individuals into two clusters, one for seedlings from Perivoli and a second from Karitsa I and II.

INSTRUCT results for K = 5 are very similar to STRUCTURE genetic groups (S5B Fig). Lowest selfing rate (0.095) was estimated for cluster IV (Ossa Massif) and highest (0.316) for cluster III (Mariolata). Values for other clusters ranged between 0.20 and 0.23 (Cluster I = 0.222, Cluster II = 0.226, Cluster V = 0.203).

**Fig 2:**
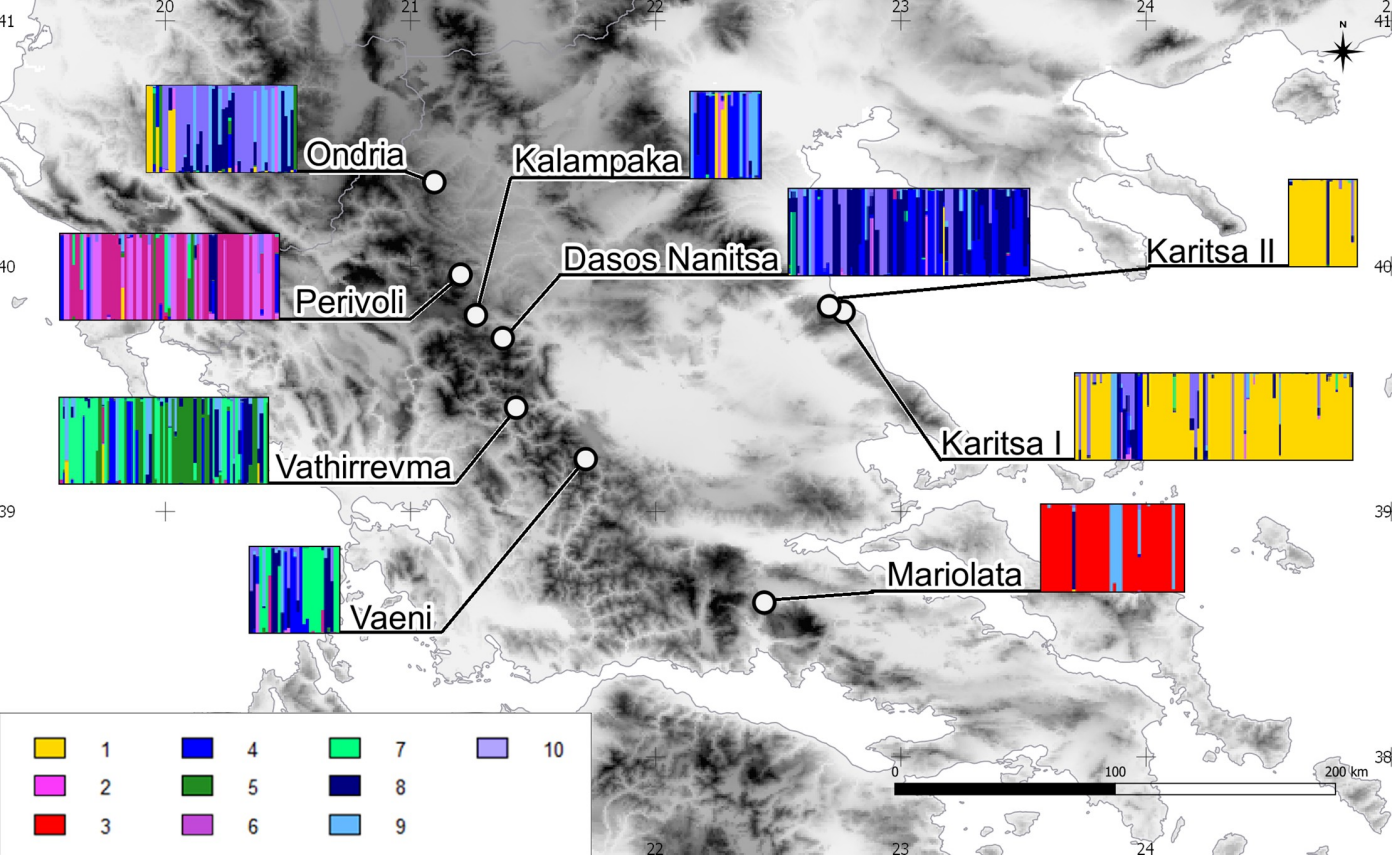
Genetic cluster inferred with DAPC analysis.

DAPC revealed a hierarchical population genetic structure among the analyzed populations, with an optimal number of clusters of K *=* 10. Among those ten clusters, five main clusters can be readily distinguished, which corresponds to results obtained with STRUCTURE (Fig 2, S7 Fig). Two clusters are present only in Perivoli indicating a cryptic substructuring in this population (cluster 2 and 6), one is specific to the isolated population of Mariolata (cluster 3) and another (cluster 1) was found in populations from Ossa Massif (Karitsa I and Karitsa II). Clusters 5 and 7 were mainly in Vathirrevma, while remaining clusters (4, 8, 9, 10) were dispersed across all the analyzed populations, with some tendency to occur in populations from the Pindos Mts. DAPC conducted for seedling populations showed that young individuals from each stand formed a separate genetic cluster.

### Isolation by ecology (IBE)

The genetic differentiation pattern was driven firstly by topography (r = 0.59, P *=* 0.0225), which partially proves IBE. Habitat suitability was not a significant factor of differentiation (r = 0.34, P *=* 0.010). The theoretical model of gene flow from CIRCUITSCAPE showed generally favorable conditions for gene flow in the core range, and pointed to impeded genetic connectivity with the most isolated population from Mariolata (S10 Fig). The genetic connectivity between the Pindos Mts. and Ossa Massif was also not affected by any physical barrier.

### Climate niche modeling

**Fig 3:**
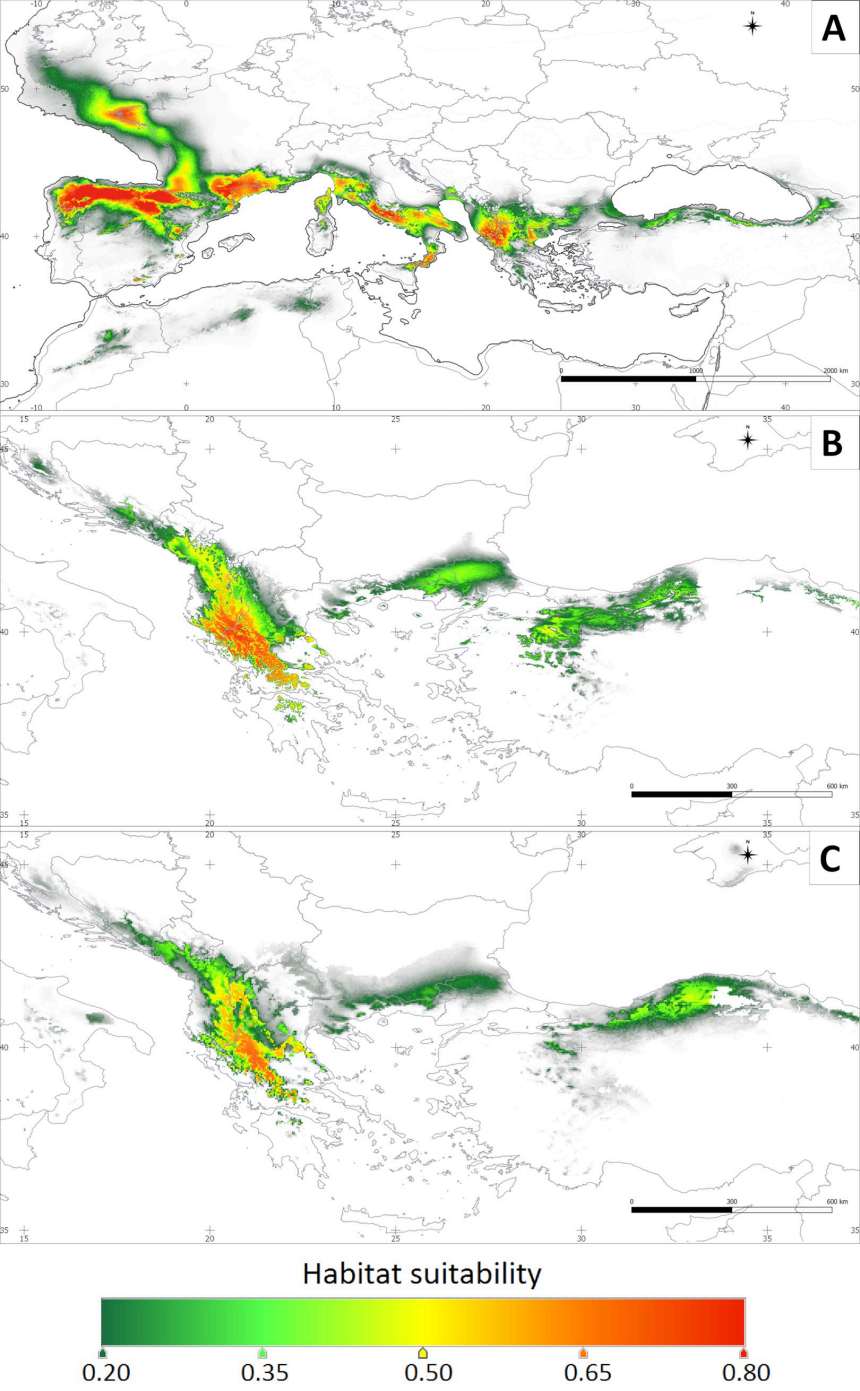
Theoretical range of *Aesculus hippocastanum*, estimated using MAXENT. A—period of the maximum glaciation (*c*. 22,000 years ago); B—current conditions; C—future conditions estimated for RCP 4.5 scenario of the climate changes.

MAXENT models based on our nine sampling locations and 65 records from the literature gave robust predictions on horse-chestnut’s past, present and future distribution (Fig 3, S8 Fig, S9 Fig). All models received AUC (Value of Area Under the Curve) greater than 0.995, indicating a very good fit of the model. The most important factors that limit the species distribution are mean temperature of the wettest quarter (relative contribution 29.0, Table 3), precipitation of the coldest quarter (21.1) and precipitation of the driest month (19.3). Quite important also are isothermality (13.9), which quantifies how large the day-to-night temperatures oscillations are relative to summer-winter temperatures, and precipitation seasonality (11.9).

**Table 3:** Percent contribution of the most important bioclimatic variables in the tested climate models.

Model	Bio8	Bio19	Bio14	Bio3	Bio15
Current conditions	29.8	18.3	20.1	14.5	12.4
LGM	30.8	19.6	19.4	13.7	11.2
Middle Holocene	29.0	22.1	19.2	13.8	11.7
Future conditions, RCP 2.6	28.8	20.3	19.6	14.0	12.5
Future conditions, RCP 4.5	27.9	23.1	18.6	13.7	11.9
Future conditions, RCP 8.5	27.9	23.1	18.6	13.7	11.9
**Average**	**29.0**	**21.1**	**19.3**	**13.9**	**11.9**

Bio8 –mean temperature of wettest quarter; Bio19 –precipitation of coldest quarter; Bio14 –precipitation of driest month; Bio3 –isothermality; Bio15 –precipitation seasonality.

The predictions of horse-chestnut’s current theoretical range mostly overlap our sampling locations from the Balkan Peninsula, especially the Pindos Mts. (suitability > 0.65). This indicates that our sampling for genetic analysis covered the core of the species potential range. There was some overprediction of the model in an eastern direction where the species does not currently occur. Specifically, the Pontic Mts. in Anatolia were highly supported as a possible area of the species distribution (Fig 3, S9A Fig).

During the period of the Last Glacial Maximum (LGM, *c*. 22,000 BP), the theoretical range of the species was likely much larger, and a westward expansion is evident (S8A Fig). Apart from the Balkan Peninsula, horse-chestnut could grow in wide areas of the northern coasts of the Mediterranean Sea and the Atlantic coast of Europe. The most suitable (>70%) were the territories by the Gulf of Lion, the Apennines, and the Atlantic coast in the northern part of the Iberian Peninsula (Cantabrian Mts.) and Brittany. In Anatolia, only a narrow stripe in the Pontic Mts. appears as a potential area of distribution but with only a low suitability (< 35%).

During the Mid-Holocene (*c*. 6000 BP), the theoretical range of horse-chestnut decreased, but it was still wider than the current range (S8B Fig). The major change was the elimination of the marginal areas delineated as less suitable during LGM (< 20%). The model also excluded areas by the Gulf of Lion, which were strongly predicted to have populations of horse-chestnut during the previous period. The main distribution during the Holocene were the Pindos Mts. on the Balkan Peninsula and the Cantabrian Mts. on the Iberian Peninsula. The western part of Anatolia appears as a suitable area (as high as 80%) despite its lack of suitability in the previous period.

In terms of future distribution, RCP 2.6 and RCP 4.5 climate changes scenarios are predicted not to have a significant impact on the central stands of horse-chestnut, although they will reduce the potential range (S9B Fig, S9C Fig). Extreme projected climate change in scenario RCP 8.5 may, however, pose a threat to the natural populations (S9D Fig). The models do not predict a change in bioclimatic conditions, which could result in a shift of the species range to the north.

## Discussion

### Level of diversity and its factors

Jiménez-Mejías et al. [44] stated that the level of genetic diversity within endemic species from the Mediterranean zone is related more with historic and environmental factors than with their biology. Because horse-chestnut is a paleoendemic and a relict with highly fragmented distribution we expected to find rather low genetic variability due to historical processes leading to range fragmentation and contraction [44]. Nevertheless, diversity assessment based on eight microsatellite loci was moderate and similar to other endemic woody species from the Mediterranean region [98–102]. However, inferences based on a small number of loci that do not represent the whole genome may be biased.

Being a Tertiary relict, horse-chestnut thrived for a long time under the relatively stable environment of climatic refugia. According to our modeling, its occurrence in Greece represents the core species distribution since at least the last glaciation, which could be an important factor in the diversity reported here [103]. Long-term survival keeps the old genetic diversity preserved and allows the new genes to accumulate [103, 104]. Recent reviews support the trend of moderate to high genetic variability among Mediterranean endemics [44, 105] and examples from other regions suggest that this may not be specific to the Mediterranean [106–111]. However, the factors underlying the retention of high genetic diversity in endemics are still rather elusive [51, 112, 113].

Over recent years, many studies have shown the high resilience of trees to negative effects of fragmentation due to their longevity, prolonged juvenile stage and high rate of gene flow. All of these are thought to retard the loss of genetic diversity even in endemic species [102, 107, 114, 115]. Hence, in the case of tree relicts, species biology can be as important as historical factors in its effect of genetic diversity. However, conclusions on high genetic variability based on mature populations may not reflect the true situation [116]. Accordingly, a closer look into differences in diversity levels between mature and seedling generations of horse-chestnut revealed some alarming symptoms of diversity leakage. Generally, two out of the three mature-offspring population pairs that were studied showed a suggested decrease in allelic richness and increased inbreeding, although differences were not statistically significant; and heterozygosity decreased in all offspring populations in comparison to mature ones (Table 2). Comparison of heterozygosity between analyzed loci show similar differences between mature and offspring populations (S3 Table); higher value of expected heterozygosity in Karitsa II–seedlings than in Karitsa II–mature probably results from a small number of analyzed individuals in mature population. Increased homozygosity in young ontogenetic stages is frequently noted, and inbreeding depression is expected to eliminate inbred individuals between seed and adult stages. However, the outflow of the allelic diversity noted in seedlings is worrying, even where the acquisition of new alleles was noted. Detection of new alleles means either that immigrants moved in, or that the new alleles were simply omitted in the mature population due to their low frequency and insufficient sampling.

In all populations of horse-chestnut investigated here (except for Dasos Nanitsa), a bottleneck was inferred. However, this estimation may be rather weak because it used only eight loci, where seven is considered a minimum in this type of analysis [117]. Considering that the range of the species has been reducing since at least last glacial cycle, a bottleneck effect ought to be assumed, although it is not a general rule among relict species [117–120]. Nevertheless, the diversity in studied populations was still maintained at a considerable level probably due to traits generally perceived as favouring high variability in tree species [19, 121, 122]. Longevity along with high fecundity would be the factors that might mitigate the effects of fragmentation on the loss of diversity [19]. High numbers of low-frequency alleles may suggest that populations experienced a less severe bottleneck and are now at the expansion phase.

### Spatial genetic structure and its factors

In principle, becoming a paleoendemic involves the shrinking and disintegration of the once widespread ancestral range [5, 123]. Distribution in isolated stands may hamper the gene flow contributing to diversity loss due to genetic drift [48, 124]. Hence, considerable genetic differentiation in this group of plants is expected since population genetic theory predicts that allele frequencies diverge among small, fragmented and isolated populations [125, 126]. Indeed, the value of *F*_*ST*_ = 0.109 indicates a moderate level of genetic differentiation, comparable to several Mediterranean conifer species that do not have such a fragmented and narrow distribution as horse-chestnut [105, 127, 128]. Another Tertiary relict, *Taxus baccata* L. that has a much wider distribution was reported to attain a very large among-population divergence [129–131]. However, English yew suffers from strong limitations of gene flow, biparental inbreeding and almost no natural regeneration [132–135]. Poor regeneration and high seedling mortality is a common problem in Mediterranean relict species, for example *Frangula alnus* Mill. subsp. *baetica* (E. Rev. and Willk.) [136], *Buxus balearica* Lam. [137] and *Olea europaea* L. subsp. *laperrinei* (Batt. and Trabut) Cif. [138]. In comparison, the demographic situation in horse-chestnut populations seems to be better since abundant natural regeneration was reported in some populations [52].

Detection of significant IBD indicates that geographical separation among horse-chestnut populations is responsible for the differentiation detected. This means that historical factors, such as range contraction induced by climatic transformations, are important drivers of spatial genetic structure in horse-chestnut. Low admixture among populations from distinct geographic regions and generally a very low rate of recent gene flow both argue for limitations in current genetic connectivity among populations, which is a consequence of range disintegration that started even before the last glaciation. This fits with the highest values of *F*_*ST*_ in pairwise comparisons being found between the most distant and marginally located populations, Mariolata and Karitsa, and the remaining populations; the lowest differentiation was noted between Karitsa I and Karitsa II separated with only *c*. 6 km.

Both, DAPC and STUCTURE indicated strong divergence of marginal populations (Mariolata, Karitsa I and II) and the very distinct character at Perivoli from the northern Pindos compared to other populations from this part of the mountain range (Fig 2). Populations from southern Pindos were also different from the northern Pindos. Thus, DAPC suggests the existence of different gene pools for horse-chestnut in the northern and southern Pindos Mts. The fact that both southern Pindos populations (Vaeni and Vathirrevma) are located by the rivers of the Ionian Sea watershed, while the northern Pindos’ rivers flow to the Aegean Sea may contribute to the observed differentiation. Results from STRUCTURE were less exact, giving Vaeni an intermediate position between Vathirrevma and the northern Pindos populations (Fig 1).

Phylogeographic studies underline the major role of southern European mountains as the centers of refugial areas during the last glacial cycle [8]. The physiographic complexity of the mountain landscapes offered shelter during the climate harshness of the Pleistocene, especially for Tertiary relicts [4, 5, 29]. However, complex mountainous landscapes may also promote genetic divergence, especially in endemics [31, 44, 139]. Indeed, our IBE analysis indicated that out of the two ecological factors considered; only topography was an important driving factor in horse-chestnut differentiation. The relatively high *F*_*ST*_ value between Mariolata and the remaining populations (average value 0.195) particularly well exemplifies the issue. Mariolata is located at the southern edge of the species distribution in the Parnassus Range, which is in the southwestern spur of the Pindos Mts. This part, unlike to the main Pindos range which runs along a north-south axis, is more latitudinally-oriented and divided into many smaller mountain ranges [140]. Such spatial orientation of ridges may produce additional obstacles for gene flow [141, 142].

Contrary to our initial assumptions, habitat characteristics were not an important factor in differentiation. We expected to find at least a weak signal of adaptive divergence since some populations inhabit ecologically very dissimilar habitats. Additionally, limited gene flow would be a factor enhancing such divergence. One of the reasons that we failed to detect local adaptation may stem from the limited number of populations included in our investigations or too small an area covered (only the Greek part of the range); only two populations out of nine analyzed showed a distinct edaphic character, being located on steep and dry slopes. For *T*. *baccata*, Mayol et al. [131] confirmed a significant influence of environmental variables on the current pattern of genetic differentiation. In *T*. *baccata*, survival in spatially isolated refugia under divergent temperature regimes were significant determinants of current genetic differentiation and consequently lead to evolution of two evolutionary lineages adapted to different temperature ranges.

Clusters inferred by STRUCTURE and INSTRUCT were related to specific mountain ranges (Fig 1). Such structuring shows that the gene flow among regions, and even between adjacent populations, is too low in such a complex environment to counteract the effects of the accumulated differentiation (Fig 1). However, gene flow is also strictly related to mechanisms of pollen and seeds dispersal. Hence, species biology may be a limiting factor for gene flow. Pollen movement distance in closely related *A*. *turbinata* may reach on average of *c*. 180 m with maximum distance reported of over 700 m. Similar distance may be attained in horse-chestnut, and it may account for effective within-population gene flow resulting in high within-population diversity. However, this distance is too short to maintain among-population connectivity in so disjunct a range and such a complex environment. Generally, in animal-pollinated tree species, highly mobile pollinators such as birds can work effectively in fragmented landscapes but in the case of insect-pollinated trees, pollen movement is mostly local [143]. Pollen of tropical tree species is moved further by insects, but even here pollen dispersal over more than a few kilometers happens only occasionally [144].

Two populations from the easternmost margins of the species range, Karitsa I and II (Ossa Massif), are separated from the core range although genetic admixture from the northern Pindos was revealed by STRUCTURE. CIRCUITSCAPE. This indicates, at least theoretically, that there are no impenetrable barriers preventing gene exchange between the Pindos Mts. and the Ossa Massif (S10 Fig), and gene flow may be asymmetric—primarily in a west-east direction (S2 Fig, S3 Fig, S10 Fig). We conjecture that this may be related to the hydrochory of species linked to the river network as the rivers from the northern Pindos that run down to the Aegean Sea. There are no direct studies on seed dispersal by water in horse-chestnut, but its populations are restricted to the watercourses which suggests the possibility [53]. In *Frangula alnus* subsp. *beatica* and other Tertiary endemics growing by mountain rivers in southern Spain, winter flooding events were shown to play an important role in the secondary dispersal of seeds already spread by birds [136]. Seeds of horse-chestnut are primary barochoric but dispersal by rodents or other animals should be considered [145, 146]. Congeneric *A*. *turbinata* is dispersed for distance of up to 114.5 m [61]. Similar dispersal distances can be expected for horse-chestnut as the seed of both species are of comparable size. However, as with wind-mediated gene flow, animal-dispersion of seeds would tend to account for local gene flow while long-distance gene transfer would require the involvement of a more effective medium; here, the mountain rivers would suit perfectly. In their work, Robledo-Arnuncio et al. [141] showed that watersheds were involved in the phylogeographic pattern of Scots pine in the Northern Meseta of Spain.

Other explanations for the genetic admixture observed between the northern Pindos and Ossa Massif and for the overall moderate value of genetic differentiation include the historically larger distribution and more extensive gene exchange in the past. Strong support for this comes from the estimations of the historical migration rate (S3 Fig) and modeling potential distributions (Fig 3). Firstly, MIGRATE-n showed more intensive gene flow among studied populations in the past. Large and demographically stable populations in the past, connected *via* gene flow and longevity would synergistically buffer horse-chestnut from the negative effects of range fragmentation for a long time. Secondly, modeling of theoretical range clearly shows a possibility of wider distribution in the past. Accordingly, during LGM (ca. 22 ka BP) *Aesculus* sp. populations might have occupied extensive areas on the west coast of the Mediterranean Sea (Fig 3). Paleodistribution modeling predicted five main domains of species occurrence which were the Pindos Mts., Apennines, areas over the Gulf of Lion, Cantabrian Mts. and the Brittany Peninsula. Except for the two latter locations, fossil data support the presence of horse-chestnut in those areas during the Pleistocene [40, 147]. Mid-Holocene sites of horse-chestnut might even have existed in south-east Ukraine [148], which was also weakly supported by our distribution modeling (1% of suitability, S4 Fig). Based on these, we propose that Pleistocene refugial populations might have existed for horse-chestnut in the Gulf of Lion, Apennines and Pindos Mts., although we cannot distinguish the existence of such population in the Cantabrian Mts. or Brittany.

Lack of palaeobotanic data on the past occurrence of horse-chestnut in Anatolia makes its presence there tentative. MAXENT projected the existence of suitable habitats for horse-chestnut in the mid-Holocene despite it being absent during the last glaciation, which is rather puzzling. Considering its limited colonization ability due to heavy seeds and the mode of their dispersion, the Holocene colonization of these areas from the Balkan Peninsula across the Aegean Sea is highly unlikely. More mysterious is the introduction route of horse-chestnut from Turkey to Europe [41]. It is possible that against the prediction of niche modeling, horse-chestnut grew in Anatolia during the LGM in local, limited refugia from which it expanded in the mid-Holocene. Unfortunately, there are no fossil records from this period and the only late Pliocene pollen record was found in eastern Anatolia [149]. It is possible, that horse-chestnut was not native to Turkey when discovered by European botanists in the 16th century. It could have been introduced to Turkey from Greece or Albania in the past, for example at a time when the Balkan Peninsula was conquered by the Ottoman Empire. However, until new paleaobotanical data are collected, neither of these hypotheses can be verified.

### Conservation remarks

Given the high fragmentation of the horse-chestnut range, there is a serious risk that the current level of divergence will be increasing since observed gene flow seems to be insufficient to maintain functional connectivity among the remnants dispersed in a complex mountainous landscape. Horse-chestnut is a habitat specialist, like other paleoendemic species [13], and may not be able to adapt to new conditions. Environmental change, human pressure and especially limitations of the species biology hinders it from rapid colonization and successful competition. Combination of these factors makes the future of this species uncertain.

Our results indicate increased homozygosity in natural populations, implying a risk of inbreeding depression and loss of diversity in the future (Table 2). In most cases, the homozygote excess was due to inbreeding, which may be accounted for by both selfing and biparental inbreeding. Although data on the mating system of horse-chestnut are missing, the conspecific *A*. *turbinata* shows a considerable level of selfing (8.3% self-pollinated seedlings) [150, 151]. Horse-chestnut is primarily entomophilous although the concentration of air-borne pollen and existence of fossil pollen records suggest that it may be partially anemophilous as well [39, 59]. The homozygosity excess could be also generated by a Wahlund effect. STRUCTURE revealed hidden population substructure in all (except one) mature populations that may suggest the existence small local breeding groups. The species reproductive biology in terms of limited capacity of pollen/seed dispersion could be the possible cause of the observed substructuring and thus, the homozygosity excess. However, this issue requires further studies.

Many species are currently at high risk from the negative impact of climate change. In the face of changing environmental conditions, endemic species may not be able to keep pace with these changes and therefore may be first to disappear [152]. For example, the range of the Chinese Tertiary relict *Davidia involucrata* Baill., is expected to be reduced to less than 30% of its current theoretical range [153]. In the Mediterranean region, the distribution of many plant species is limited by water availability [5, 154, 155] and drought is one of the most important factor that leads to the decline of forest in this region [156]. Future climate change in the Mediterranean may bring some risk to natural populations of horse-chestnut, mainly by the reduction of rainfall and increasing summer temperature [157]. According to future range modeling, a moderate scenario of climate change should not cause extinction of the populations in the Pindos Mts., but extreme changes (RCP 8.5 scenario) entails some risk for species survival.

The results obtained in this work are less optimistic about the future persistence of horse-chestnut than previous studies [52]. The most important contributing factors are also different. These result from using a more accurate model. However, it should be remembered that modeling provides only estimations and the differences between various models can be large, especially when considering future conditions [158, 159]. Unfortunately, current models of the future range of horse-chestnut do not show a large expansion of this species to the north to trace the optimum conditions. Thus, *in situ* conservation should be a priority and a conservation program should be immediately launched for Greek populations, which are the largest [49]. Unfortunately, as observed during this study, most of the natural populations are not protected and some of them are being destroyed by infrastructure expansion. Although the most valuable natural populations of the horse-chestnut are located in Greece, this species is not on the Red List of Greece and a reasonable conservation strategy is lacking [160]. So far horse-chestnut has been included in the national list of protected species of the Presidential Degree 67/1981, and some populations are within the NATURA 2000 network [38], but it is not enough. Much more needs to be done and surely can be done in order to protect this beautiful tree in its natural environment.

## Supporting information

S1 Fig Optimal number of clusters based on Evanno's delta K. (TIFF)

S2 Fig Theoretical current gene flow between populations estimated with BAYESASS and visualized using QGIS. (TIFF)

S3 Fig Theoretical historical gene flow between populations estimated with MIGRATE-N and visualized using QGIS. (TIFF)

S4 Fig Neighbor-joining tree based on Cavalli-Sforza and Edwards genetic distances for clusters determined by STRUCTURE. (TIFF)

S5 Fig Results of STRUCTURE and INSTRUCT analyzes. A—comparison between genetic clusters determined by STRUCTURE (left, all mature populations included in analysis, best K = 5) and substructure of each population (right, separate analysis for each population). B—comparison between STRUCTURE and INSTRUCT results.
(TIFF)

S6 Fig Best K for each STRUCTURE analyze for separate population, according to ln(Pr(X|K) values. (TIFF)

S7 Fig Ordination plot for the first two discriminant axes obtained DAPC analysis. (TIFF)

S8 Fig Theoretical range of *Aesculus hippocastanum* in the past. A—period of the maximum glaciation (ca. 22,000 years ago); dark, bold line indicate a coastline during the maximum glaciation period; B—Mid-Holocene (ca. 6,000 years ago).
(TIFF)

S9 Fig Current and future theoretical range of *Aesculus hippocastanum*. A—theoretical range in current conditions; B—future theoretical range estimated for RCP 2.6 scenario of the climate changes; C—future theoretical range estimated for RCP 4.5 scenario of the climate changes; D—future theoretical range, estimated for RCP 8.5 scenario of the climate changes.
(TIFF)

S10 Fig Theoretical gene flow in relation to environmental conditions determined using CIRCUITSCAPE. Lighter colors means best ways for gene flow (higher conductance); darker colors—barriers for gene flow (lower conductance). Populations are shown as red cylinders. Blue boxes show cities.
(TIFF)

S1 Table Geographical coordinates and bioclimatic variables of 74 natural populations of horse-chestnut used in MAXENT analyze. Coordinates for populations from Peçi et al. (2012) are approximate.
(XLSX)

S2 Table Bioclimatic variables from WorldClim database. Variables used in the analysis of the theoretical range are bolded.
(DOCX)

S3 Table Parameters of genetic diversity of analyzed loci. A–number of alleles, A_e_−effective number of alleles, Null–frequency of null alleles, Ho−observed heterozygosity, H_e_−expected heterozygosity.
(XLSX)

S4 Table Matrix of genetic distance between populations. F_ST_ with ENA correction above diagonal, F_ST_ without ENA correction below diagonal. Populations: 1 –Ondria, 2 –Kalampaka, 3 –Dasos Nanitsa, 4 –Vaeni, 5 –Mariolata, 6 –Karitsa I, 7 –Karitsa II, 8 –Vathirrevma, 9 –Perivoli. F_ST_ is not significant (p > 0.05).
(DOCX)

S5 Table Matrix of migration rate between populations as calculated by BAYEASS. In rows are the source populations, in columns—the sink populations. Proportion of migrants above 2% are bolded. Populations: 1 –Ondria, 2 –Kalampaka, 3 –Dasos Nanitsa, 4 –Vaeni, 5 –Mariolata, 6 –Karitsa I, 7 –Karitsa II, 8 –Vathirrevma, 9 –Perivoli.
(DOCX)

S6 Table Matrix of migration (number of individuals per generation) between populations as calculated by MIGRATE-n. In rows are the source populations, in columns—the populations into which individuals immigrate. Number of migrants above 150 are bolded. Populations: 1 –Ondria, 2 –Kalampaka, 3 –Dasos Nanitsa, 4 –Vaeni, 5 –Mariolata, 6 –Karitsa I, 7 –Karitsa II, 8 –Vathirrevma, 9 –Perivoli.
(DOCX)

S7 Table Probability of belonging of each individual to each cluster according to STRUCTURE analysis. (DOCX)
