# Supplementary figures and images for: Spatial genetic structure and diversity of natural populations of Aesculus hippocastanum L. in Greece
Source: PLoS One. 2019 Dec 11;14(12):e0226225. doi: 10.1371/journal.pone.0226225 (PMC6905551; doi:10.1371/journal.pone.0226225)

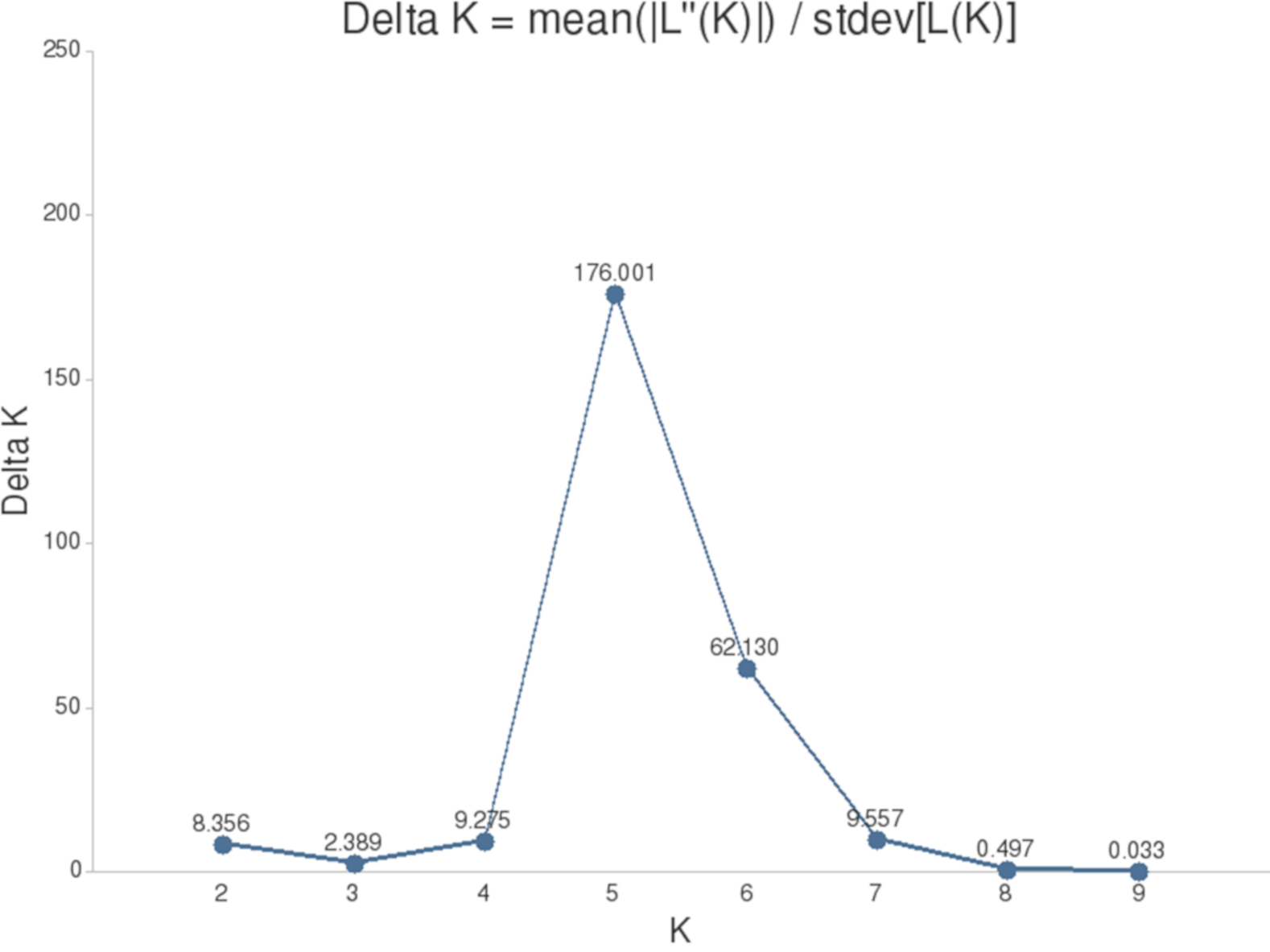

Supplement: S1 Fig — (TIFF) [file pone.0226225.s001.tiff]

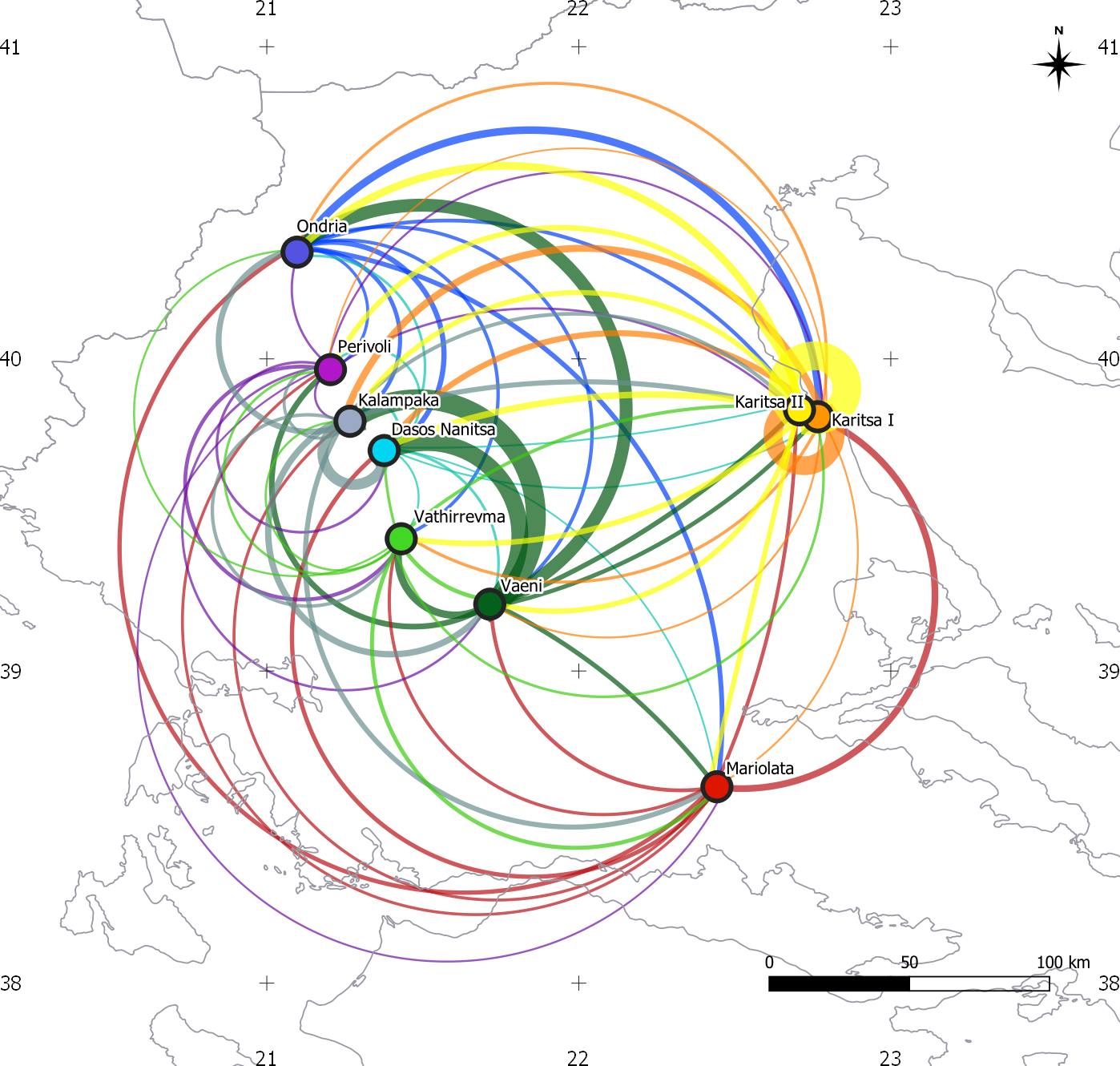

Supplement: S2 Fig — (TIFF) [file pone.0226225.s002.tiff]

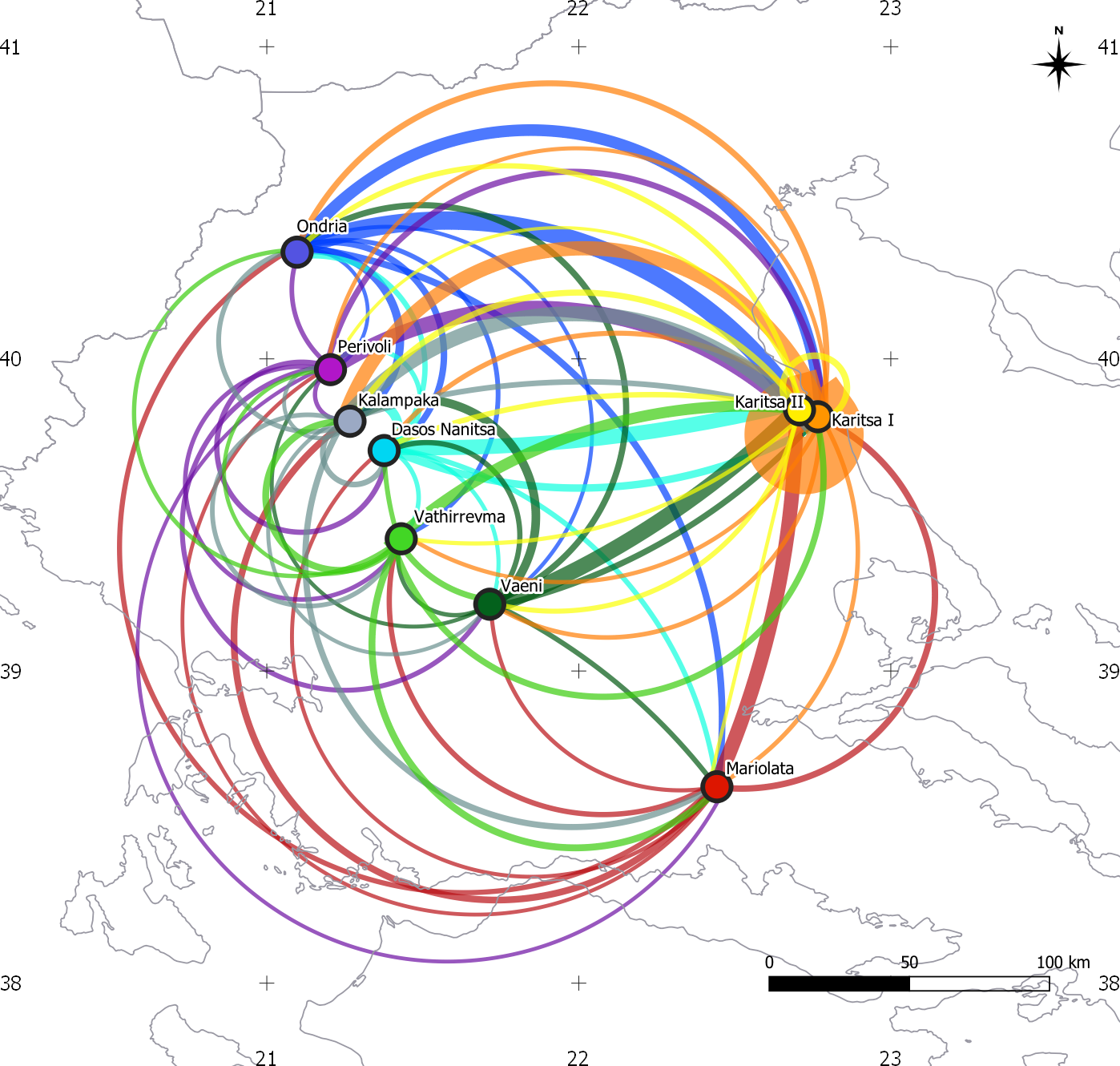

Supplement: S3 Fig — (TIFF) [file pone.0226225.s003.tiff]

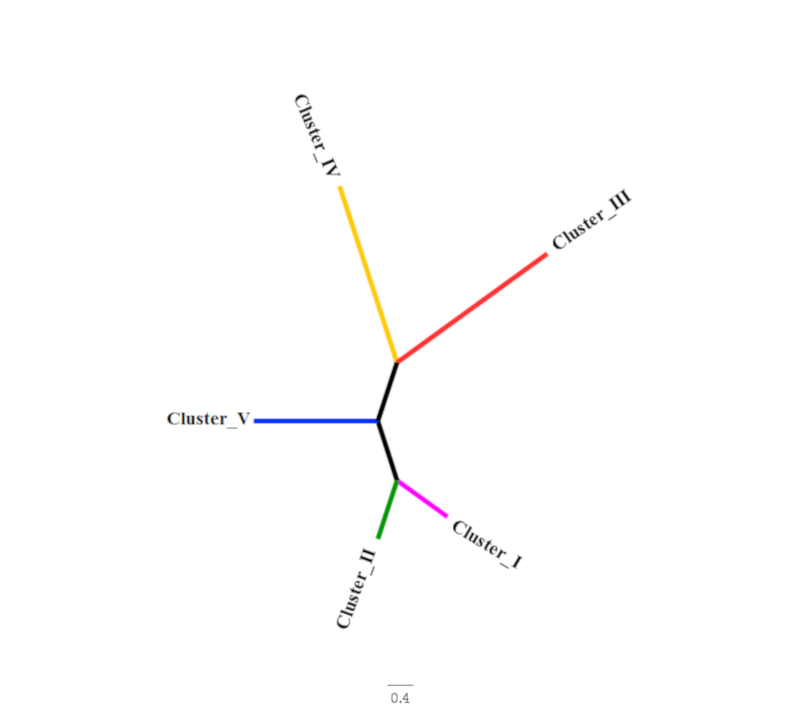

Supplement: S4 Fig — (TIFF) [file pone.0226225.s004.tiff]

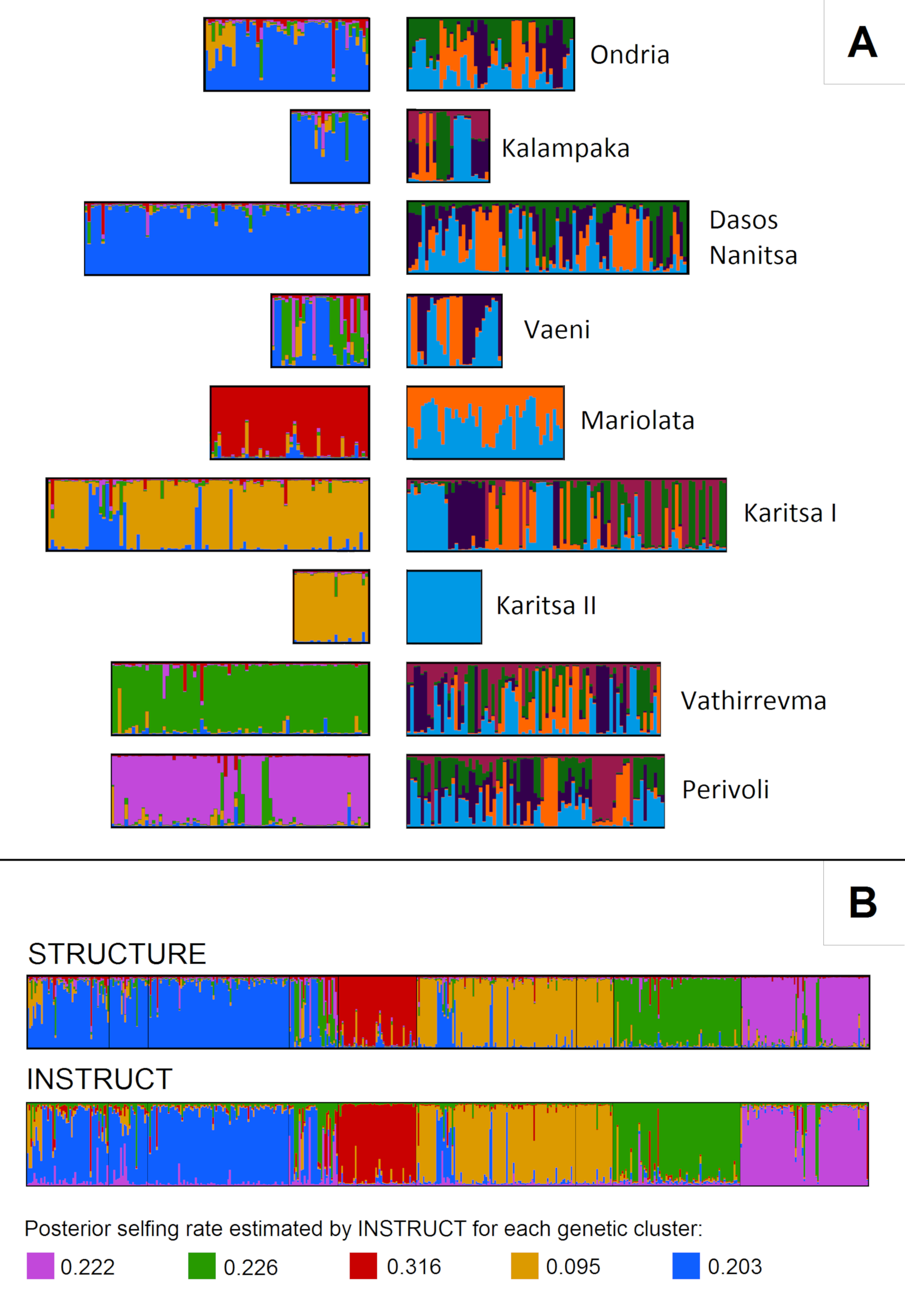

Supplement: S5 Fig — A—comparison between genetic clusters determined by STRUCTURE (left, all mature populations included in analysis, best K = 5) and substructure of each population (right, separate analysis for each population). B—comparison between STRUCTURE and INSTRUCT results. (TIFF) [file pone.0226225.s005.tiff]

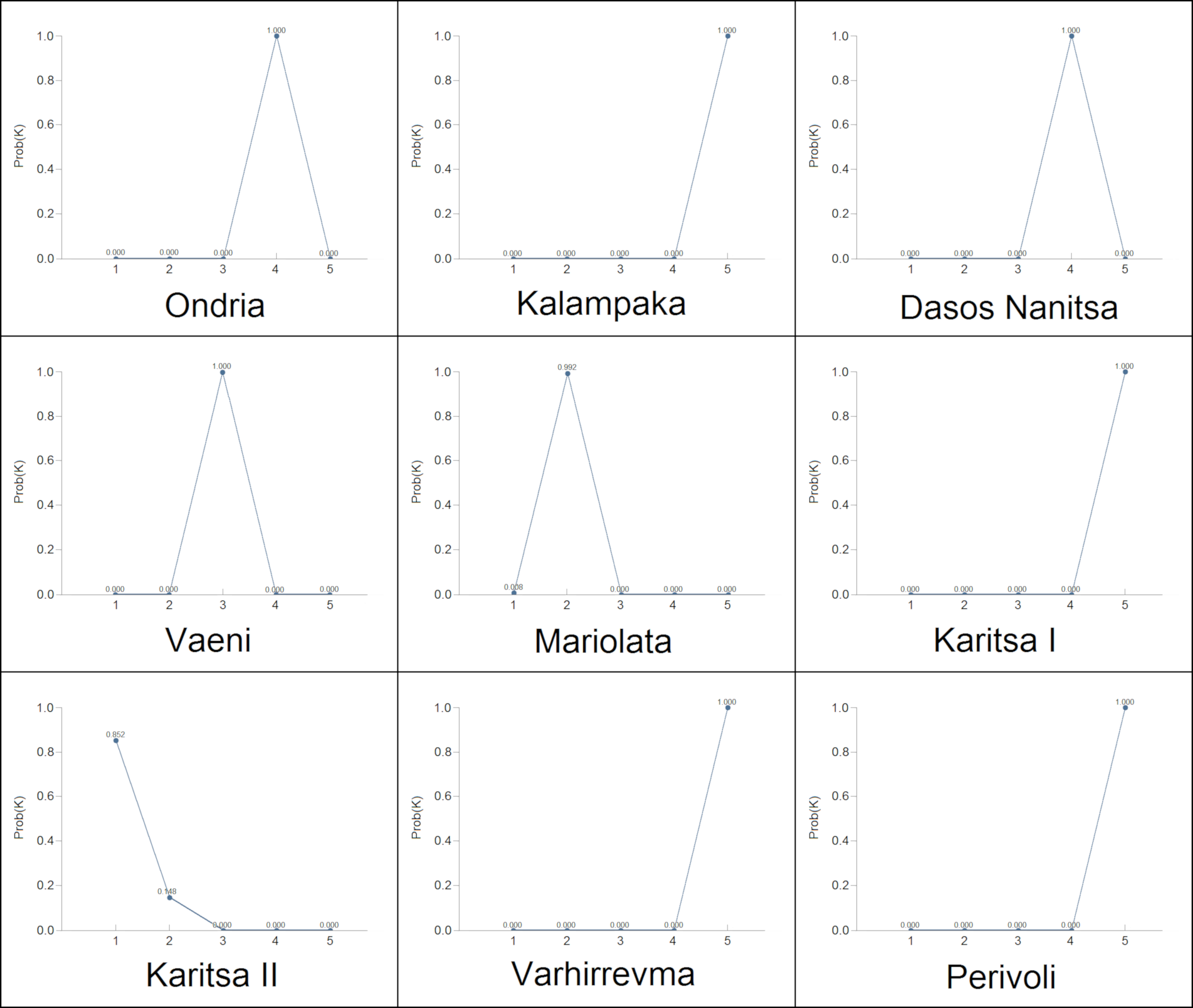

Supplement: S6 Fig — (TIFF) [file pone.0226225.s006.tiff]

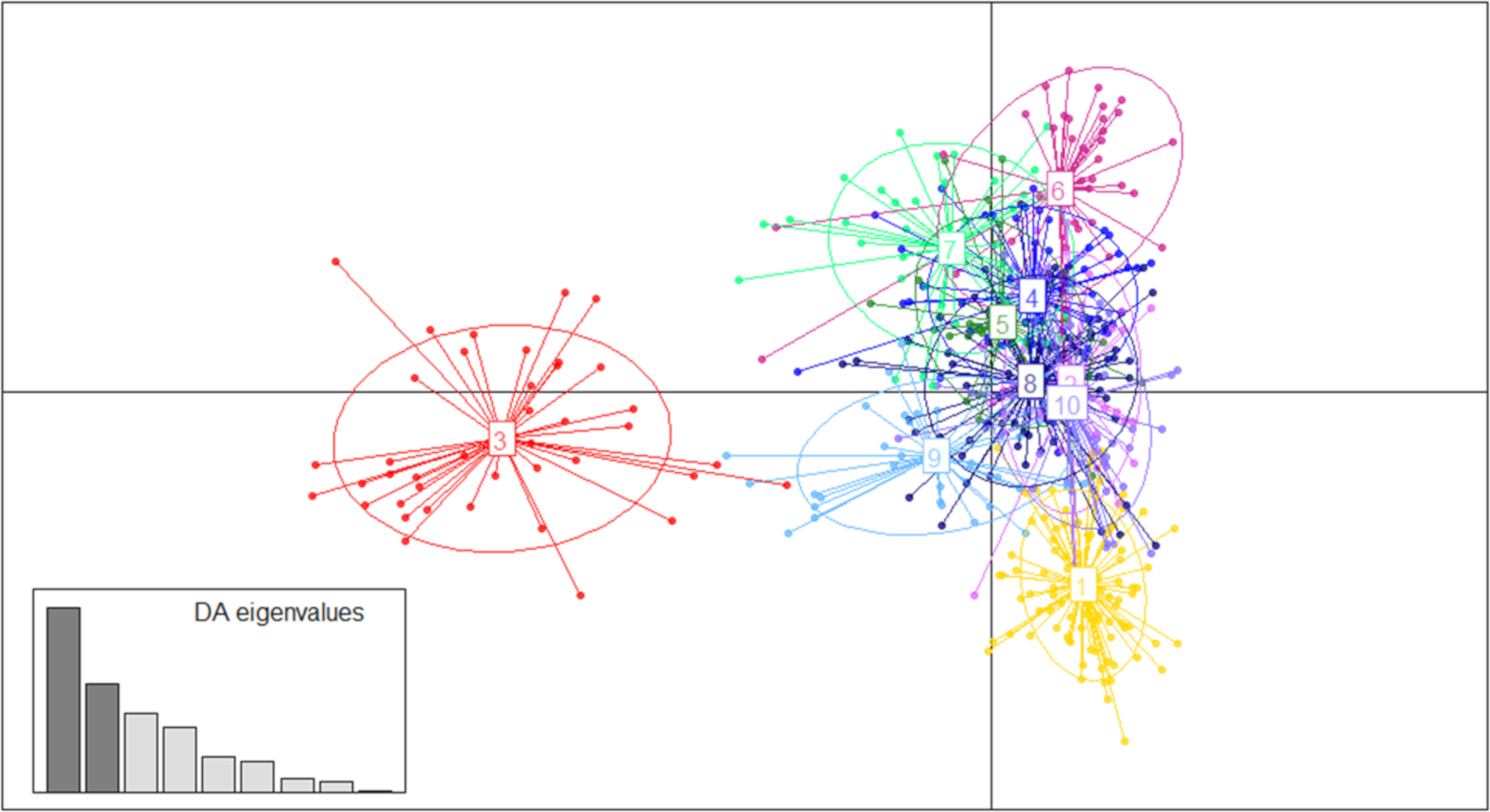

Supplement: S7 Fig — (TIFF) [file pone.0226225.s007.tiff]

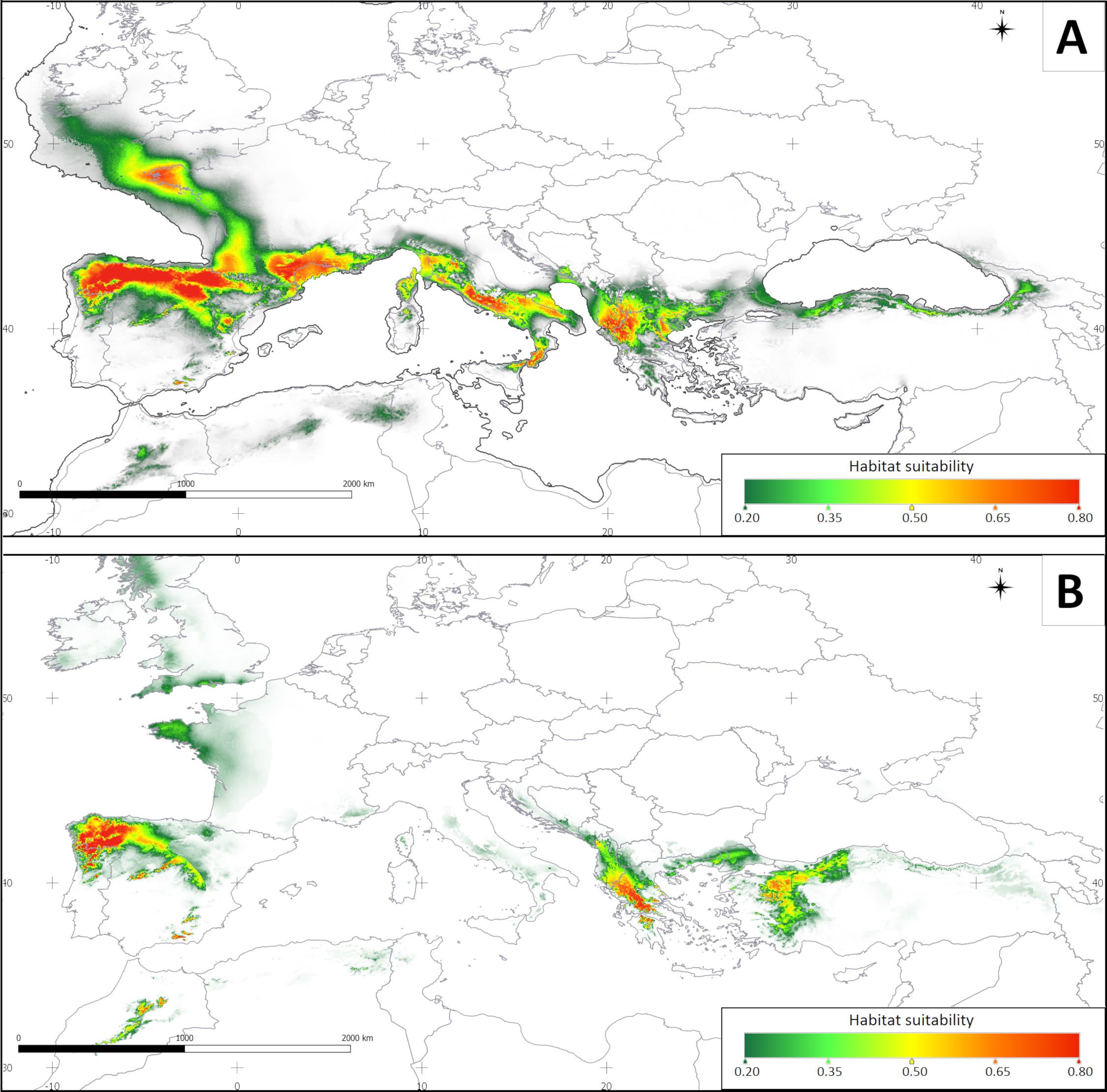

Supplement: S8 Fig — A—period of the maximum glaciation (ca. 22,000 years ago); dark, bold line indicate a coastline during the maximum glaciation period; B—Mid-Holocene (ca. 6,000 years ago). (TIFF) [file pone.0226225.s008.tiff]

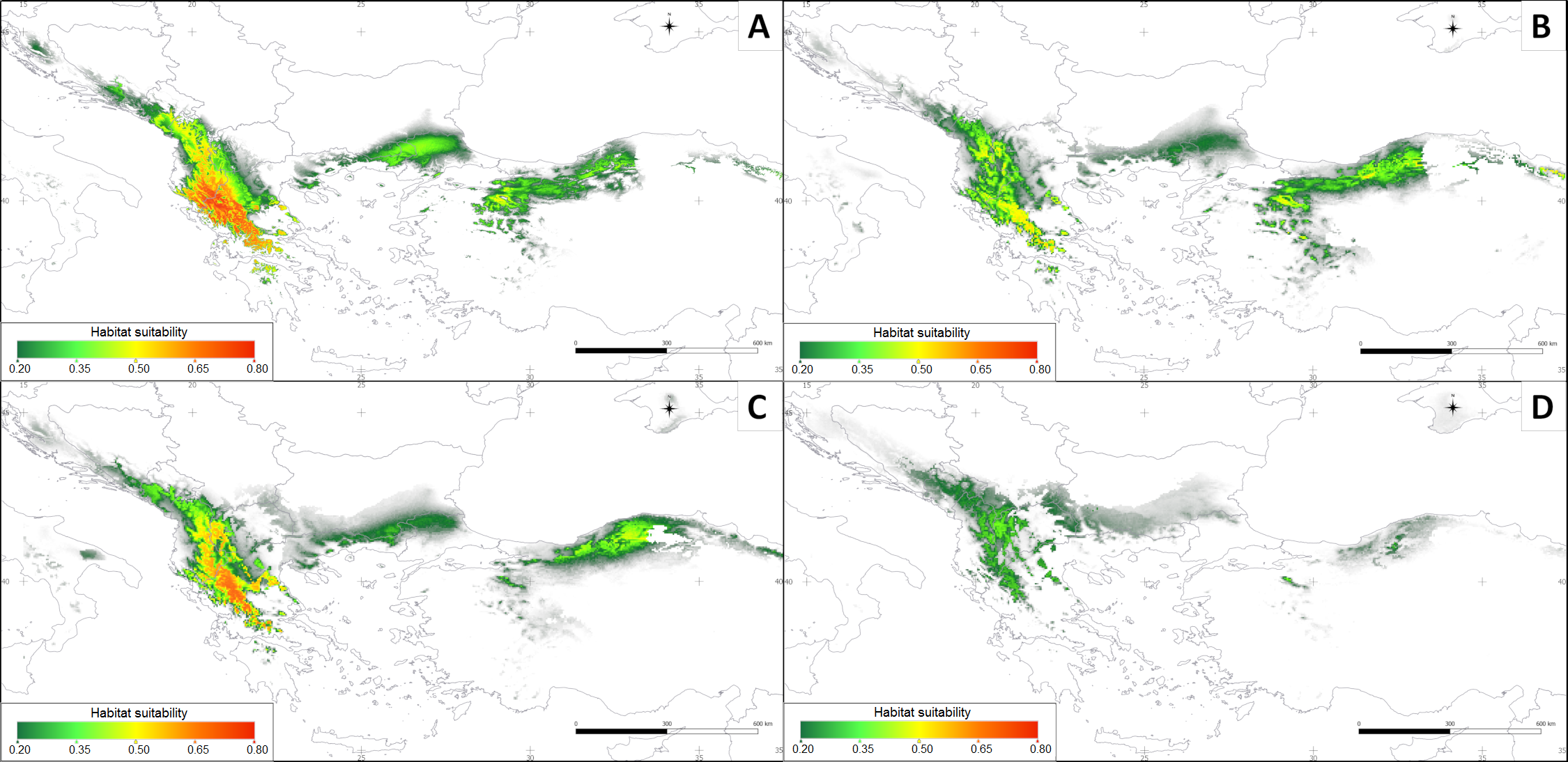

Supplement: S9 Fig — A—theoretical range in current conditions; B—future theoretical range estimated for RCP 2.6 scenario of the climate changes; C—future theoretical range estimated for RCP 4.5 scenario of the climate changes; D—future theoretical range, estimated for RCP 8.5 scenario of the climate changes. (TIFF) [file pone.0226225.s009.tiff]

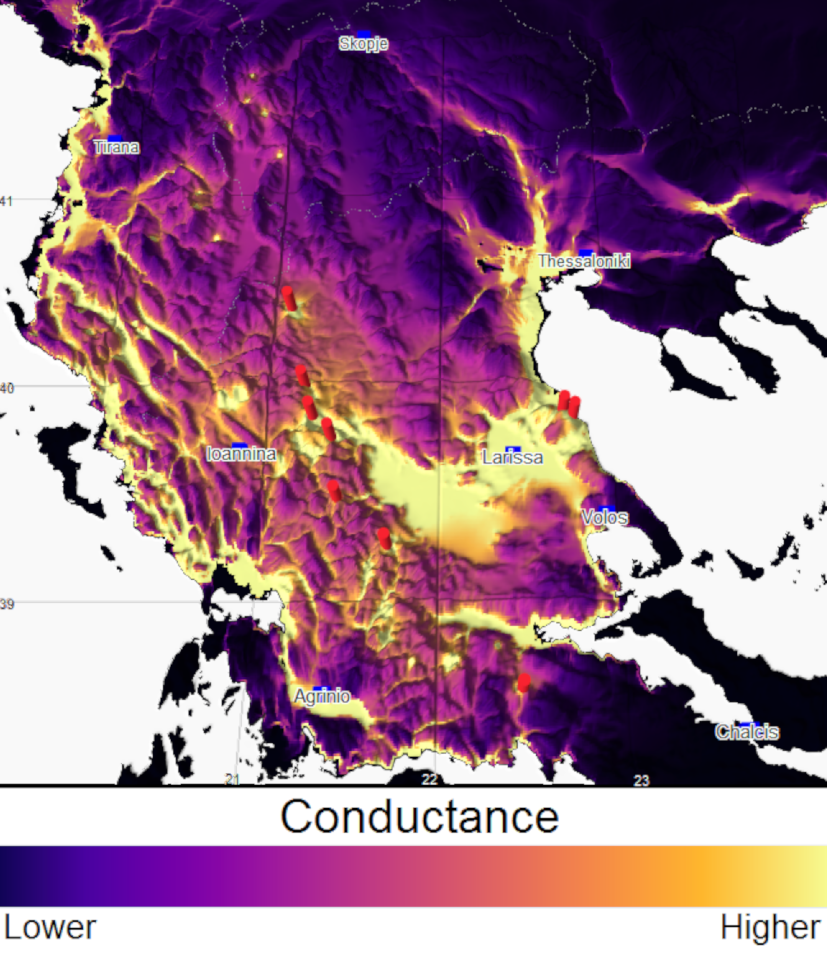

Supplement: S10 Fig — Lighter colors means best ways for gene flow (higher conductance); darker colors—barriers for gene flow (lower conductance). Populations are shown as red cylinders. Blue boxes show cities. (TIFF) [file pone.0226225.s010.tiff]
