# Supplementary material for: Spatial genetic structure and diversity of natural populations of Aesculus hippocastanum L. in Greece
Source: PLoS One. 2019 Dec 11;14(12):e0226225. doi: 10.1371/journal.pone.0226225 (PMC6905551; doi:10.1371/journal.pone.0226225)
Supplement: S2 Table — Variables used in the analysis of the theoretical range are bolded. (DOCX) [file pone.0226225.s012.docx]

| **Code** | **Variable** |
| --- | --- |
| **bio1** | **Annual mean temperature** |
| **bio2** | **Mean diurnal range = mean of monthly (max temp-min temp)** |
| **bio3** | **Isothermality (bio2/bio7) (*100)** |
| **bio4** | **Temperature seasonality (standard deviation *100)** |
| **bio5** | **Max temperature of warmest month** |
| bio6 | Min temperature of coldest month |
| bio7 | Temperature annual range (bio5-bio6) |
| **bio8** | **Mean temperature of wettest quarter** |
| bio9 | Mean temperature of driest quarter |
| bio10 | Mean temperature of warmest quarter |
| bio11 | Mean temperature of coldest quarter |
| **bio12** | **Annual precipitation** |
| **bio13** | **Precipitation of wettest month** |
| **bio14** | **Precipitation of driest month** |
| **bio15** | **Precipitation seasonality (coefficient of variation)** |
| bio16 | Precipitation of wettest quarter |
| bio17 | Precipitation of driest quarter |
| **bio18** | **Precipitation of warmest quarter** |
| **bio19** | **Precipitation of coldest quarter** |
