# Supplementary material for: Spatial genetic structure and diversity of natural populations of Aesculus hippocastanum L. in Greece
Source: PLoS One. 2019 Dec 11;14(12):e0226225. doi: 10.1371/journal.pone.0226225 (PMC6905551; doi:10.1371/journal.pone.0226225)
Supplement: S4 Table — FST with ENA correction above diagonal, FST without ENA correction below diagonal. Populations: 1 –Ondria, 2 –Kalampaka, 3 –Dasos Nanitsa, 4 –Vaeni, 5 –Mariolata, 6 –Karitsa I, 7 –Karitsa II, 8 –Vathirrevma, 9 –Perivoli. FST is not significant (p > 0.05). (DOCX) [file pone.0226225.s014.docx]

| Pop. | Ondria | Kalampaka | Dasos Nanitsa | Vaeni | Mariolata | Karitsa_I | Karitsa_II | Vathirrevma | Perivoli |
| --- | --- | --- | --- | --- | --- | --- | --- | --- | --- |
| Ondria |  | 0.081 | 0.057 | 0.067 | 0.164 | 0.067 | 0.105 | 0.083 | 0.123 |
| Kalampaka | 0.091 |  | 0.074 | 0.087 | 0.181 | 0.096 | 0.145 | 0.084 | 0.123 |
| Dasos Nanitsa | 0.055 | 0.083 |  | 0.057 | 0.191 | 0.074 | 0.105 | 0.062 | 0.102 |
| Vaeni | 0.063 | 0.095 | 0.055 |  | 0.175 | 0.086 | 0.098 | 0.046 | 0.080 |
| Mariolata | 0.179 | 0.199 | 0.201 | 0.185 |  | 0.176 | 0.227 | 0.156 | 0.214 |
| Karitsa_I | 0.069 | 0.110 | 0.077 | 0.087 | 0.186 |  | 0.035 | 0.078 | 0.107 |
| Karitsa_II | 0.111 | 0.160 | 0.114 | 0.108 | 0.239 | 0.036 |  | 0.095 | 0.129 |
| Vathirrevma | 0.081 | 0.093 | 0.063 | 0.051 | 0.165 | 0.079 | 0.100 |  | 0.083 |
| Perivoli | 0.117 | 0.128 | 0.101 | 0.084 | 0.221 | 0.104 | 0.131 | 0.090 |  |
