# Supplementary material for: Spatial genetic structure and diversity of natural populations of Aesculus hippocastanum L. in Greece
Source: PLoS One. 2019 Dec 11;14(12):e0226225. doi: 10.1371/journal.pone.0226225 (PMC6905551; doi:10.1371/journal.pone.0226225)
Supplement: S5 Table — In rows are the source populations, in columns—the sink populations. Proportion of migrants above 2% are bolded. Populations: 1 –Ondria, 2 –Kalampaka, 3 –Dasos Nanitsa, 4 –Vaeni, 5 –Mariolata, 6 –Karitsa I, 7 –Karitsa II, 8 –Vathirrevma, 9 –Perivoli. (DOCX) [file pone.0226225.s015.docx]

| Pop. | Ondria | Kalampaka | Dasos Nanitsa | Vaeni | Mariolata | Karitsa_I | Karitsa_II | Vathirrevma | Perivoli |
| --- | --- | --- | --- | --- | --- | --- | --- | --- | --- |
| Ondria |  | 0.0087 | 0.0109 | 0.0074 | 0.0101 | 0.0159 | 0.0079 | 0.0072 | 0.0064 |
| Kalampaka | 0.0124 |  | **0.0225** | 0.0134 | 0.0107 | 0.0108 | 0.0106 | 0.0152 | 0.0104 |
| Dasos Nanitsa | 0.0054 | 0.0057 |  | 0.0056 | 0.0037 | 0.0039 | 0.0039 | 0.0041 | 0.0051 |
| Vaeni | **0.0273** | **0.0419** | **0.0354** |  | 0.0098 | 0.0090 | 0.0103 | 0.0155 | 0.0120 |
| Mariolata | 0.0091 | 0.0063 | 0.0091 | 0.0080 |  | 0.0141 | 0.0078 | 0.0068 | 0.0061 |
| Karitsa_I | 0.0062 | 0.0149 | 0.0125 | 0.0042 | 0.0040 |  | **0.0393** | 0.0065 | 0.0036 |
| Karitsa_II | 0.0162 | 0.0110 | 0.0138 | 0.0111 | 0.0106 | 0.0601 |  | 0.0116 | 0.0113 |
| Vathirrevma | 0.0043 | 0.0047 | 0.0058 | 0.0052 | 0.0088 | 0.0056 | 0.0072 |  | 0.0052 |
| Perivoli | 0.0049 | 0.0047 | 0.0056 | 0.0051 | 0.0041 | 0.0044 | 0.0048 | 0.0080 |  |
