# Supplementary material for: Spatial genetic structure and diversity of natural populations of Aesculus hippocastanum L. in Greece
Source: PLoS One. 2019 Dec 11;14(12):e0226225. doi: 10.1371/journal.pone.0226225 (PMC6905551; doi:10.1371/journal.pone.0226225)
Supplement: S6 Table — In rows are the source populations, in columns—the populations into which individuals immigrate. Number of migrants above 150 are bolded. Populations: 1 –Ondria, 2 –Kalampaka, 3 –Dasos Nanitsa, 4 –Vaeni, 5 –Mariolata, 6 –Karitsa I, 7 –Karitsa II, 8 –Vathirrevma, 9 –Perivoli. (DOCX) [file pone.0226225.s016.docx]

| Pop. | Ondria | Kalampaka | Dasos Nanitsa | Vaeni | Mariolata | Karitsa_I | Karitsa_II | Vathirrevma | Perivoli |
| --- | --- | --- | --- | --- | --- | --- | --- | --- | --- |
| Ondria | 0.00 | **173.69** | 136.15 | 82.91 | 143.28 | **246.88** | **387.07** | 100.80 | 78.80 |
| Kalampaka | 103.67 | 0.00 | 118.55 | 83.20 | 130.25 | 118.09 | **317.17** | 99.36 | 82.80 |
| Dasos Nanitsa | 113.36 | **197.51** | 0.00 | 87.49 | 108.75 | **157.78** | **317.49** | 95.58 | 100.61 |
| Vaeni | 130.59 | **182.24** | 111.09 | 0.00 | 107.24 | 127.11 | **329.79** | 92.48 | 84.09 |
| Mariolata | 108.06 | 141.76 | 82.99 | 87.26 | 0.00 | 134.02 | **307.40** | 112.49 | 80.50 |
| Karitsa_I | 125.68 | **300.89** | 106.01 | 97.33 | 95.38 | 0.00 | **1202.21** | 89.35 | 85.51 |
| Karitsa_II | 113.60 | 128.57 | 114.52 | 105.95 | 77.66 | 137.33 | 0.00 | 87.64 | 68.62 |
| Vathirrevma | 91.53 | 143.40 | 101.59 | 127.70 | 145.66 | 133.13 | **234.69** | 0.00 | 82.89 |
| Perivoli | 98.85 | 92.00 | 111.93 | 105.34 | 81.46 | 128.24 | **323.68** | 93.96 | 0.00 |
