# Supplementary material for: Spatial genetic structure and diversity of natural populations of Aesculus hippocastanum L. in Greece
Source: PLoS One. 2019 Dec 11;14(12):e0226225. doi: 10.1371/journal.pone.0226225 (PMC6905551; doi:10.1371/journal.pone.0226225)
Supplement: S7 Table — (DOCX) [file pone.0226225.s017.docx]

| **No. of individual** | No. of population | Cluster I | Cluster II | Cluster III | Cluster IV | Cluster V |
| --- | --- | --- | --- | --- | --- | --- |
| **1** | Ondria | 1.23% | 0.89% | 0.40% | 41.43% | **56.06%** |
| **2** | Ondria | 1.90% | 1.42% | 5.18% | **64.21%** | 27.29% |
| **3** | Ondria | 1.20% | 27.08% | 3.50% | 37.32% | 30.92% |
| **4** | Ondria | 1.48% | 1.14% | 0.82% | 37.93% | **58.57%** |
| **5** | Ondria | 5.38% | 16.68% | 1.08% | 35.65% | 41.25% |
| **6** | Ondria | 3.62% | 3.78% | 5.34% | 23.00% | **64.24%** |
| **7** | Ondria | 2.08% | 0.90% | 1.61% | 33.92% | **61.46%** |
| **8** | Ondria | 1.59% | 0.80% | 0.40% | 36.63% | **60.55%** |
| **9** | Ondria | 4.77% | 3.46% | 1.40% | **56.86%** | 33.48% |
| **10** | Ondria | 2.50% | 0.60% | 0.50% | 0.70% | **95.61%** |
| **11** | Ondria | 3.32% | 0.70% | 0.80% | 0.64% | **94.54%** |
| **12** | Ondria | 0.91% | 0.69% | 0.60% | 0.60% | **97.20%** |
| **13** | Ondria | 2.44% | 3.21% | 17.25% | 1.30% | **75.83%** |
| **14** | Ondria | 0.50% | 0.50% | 0.50% | 2.19% | **96.29%** |
| **15** | Ondria | 2.30% | 1.78% | 0.90% | 3.14% | **91.86%** |
| **16** | Ondria | 0.90% | 2.29% | 31.92% | 4.62% | **60.28%** |
| **17** | Ondria | 16.31% | **54.55%** | 11.80% | 3.57% | 13.76% |
| **18** | Ondria | 0.97% | 0.90% | 1.85% | 0.91% | **95.32%** |
| **19** | Ondria | 1.27% | 0.60% | 1.10% | 8.33% | **88.69%** |
| **20** | Ondria | 3.09% | 2.28% | 1.12% | 1.79% | **91.75%** |
| **21** | Ondria | 0.80% | 2.06% | 3.32% | 1.30% | **92.53%** |
| **22** | Ondria | 5.95% | 1.58% | 3.01% | 7.02% | **82.44%** |
| **23** | Ondria | 7.63% | 1.11% | 6.60% | 1.28% | **83.37%** |
| **24** | Ondria | 3.36% | 2.94% | 10.15% | 4.42% | **79.15%** |
| **25** | Ondria | 1.09% | 19.96% | 1.80% | 0.53% | **76.62%** |
| **26** | Ondria | 0.80% | 4.28% | 3.14% | 0.60% | **91.18%** |
| **27** | Ondria | 0.70% | 3.99% | 1.61% | 29.58% | **64.06%** |
| **28** | Ondria | 10.56% | 3.37% | 3.12% | 6.42% | **76.52%** |
| **29** | Ondria | 13.43% | 2.23% | 4.64% | 3.31% | **76.38%** |
| **30** | Ondria | 0.80% | 2.27% | 1.61% | 0.80% | **94.48%** |
| **31** | Ondria | 2.10% | 0.50% | 0.51% | 0.90% | **95.95%** |
| **32** | Ondria | 2.00% | 0.50% | 0.50% | 0.70% | **96.20%** |
| **33** | Ondria | 2.30% | 0.80% | 0.70% | 0.90% | **95.30%** |
| **34** | Ondria | 3.45% | 1.90% | 0.60% | 1.10% | **92.95%** |
| **35** | Ondria | 0.91% | 1.90% | 0.60% | 1.47% | **95.15%** |
| **36** | Ondria | 4.00% | 1.30% | 0.70% | 5.98% | **88.04%** |
| **37** | Ondria | 0.67% | 1.13% | 0.60% | 2.17% | **95.43%** |
| **38** | Ondria | 8.42% | 3.86% | **69.55%** | 6.35% | 11.81% |
| **39** | Ondria | 0.51% | 9.52% | 2.89% | 2.31% | **84.73%** |
| **40** | Ondria | 0.80% | 0.70% | 0.60% | 1.20% | **96.70%** |
| **41** | Ondria | 0.62% | 4.39% | 2.69% | 1.00% | **91.35%** |
| **42** | Ondria | 36.66% | 1.20% | 4.61% | 0.80% | **56.75%** |
| **43** | Ondria | 2.44% | 16.43% | 4.67% | 1.50% | **74.96%** |
| **44** | Ondria | 3.83% | 4.69% | 1.80% | 2.13% | **87.58%** |
| **45** | Ondria | 2.05% | 20.55% | 2.50% | 12.00% | **62.90%** |
| **46** | Ondria | 0.60% | 1.76% | 12.52% | 0.94% | **84.23%** |
| **47** | Ondria | 2.29% | 1.62% | 23.24% | 18.86% | **53.97%** |
| **48** | Ondria | 9.69% | 36.61% | 2.71% | 15.54% | 35.43% |
| **49** | Kalampaka | 0.60% | 0.70% | 0.90% | 1.09% | **96.70%** |
| **50** | Kalampaka | 0.90% | 0.80% | 0.90% | 0.50% | **96.91%** |
| **51** | Kalampaka | 1.20% | 11.21% | 0.70% | 0.70% | **86.16%** |
| **52** | Kalampaka | 4.46% | 1.00% | 0.80% | 0.50% | **93.26%** |
| **53** | Kalampaka | 1.55% | 1.00% | 2.40% | 0.70% | **94.37%** |
| **54** | Kalampaka | 4.52% | 3.79% | 0.81% | 2.72% | **88.14%** |
| **55** | Kalampaka | 1.69% | 1.26% | 1.70% | 0.70% | **94.70%** |
| **56** | Kalampaka | 14.93% | 7.63% | 4.04% | 26.39% | 47.03% |
| **57** | Kalampaka | 5.84% | 2.27% | 0.50% | 14.10% | **77.28%** |
| **58** | Kalampaka | 35.55% | 0.70% | 17.26% | 10.81% | 35.68% |
| **59** | Kalampaka | 9.43% | 0.80% | 3.13% | 16.05% | **70.59%** |
| **60** | Kalampaka | 2.31% | 1.63% | 3.06% | 19.62% | **73.40%** |
| **61** | Kalampaka | 1.94% | 26.11% | 0.50% | 2.42% | **69.06%** |
| **62** | Kalampaka | 1.00% | 1.09% | 0.60% | 0.50% | **96.84%** |
| **63** | Kalampaka | 6.87% | 6.21% | 1.60% | 1.20% | **84.18%** |
| **64** | Kalampaka | 0.74% | 11.18% | 2.30% | 0.60% | **85.18%** |
| **65** | Kalampaka | 0.54% | **68.01%** | 1.20% | 0.60% | 29.72% |
| **66** | Kalampaka | 0.60% | 0.80% | 0.59% | 0.52% | **97.49%** |
| **67** | Kalampaka | 0.60% | 0.84% | 0.80% | 0.60% | **97.10%** |
| **68** | Kalampaka | 0.90% | 1.80% | 0.80% | 1.90% | **94.63%** |
| **69** | Kalampaka | 1.00% | 1.40% | 1.20% | 1.49% | **94.92%** |
| **70** | Kalampaka | 0.90% | 3.67% | 2.76% | 5.39% | **87.25%** |
| **71** | Kalampaka | 4.16% | 4.95% | 0.80% | 0.80% | **89.33%** |
| **72** | Dasos Nanitsa | 2.25% | 6.50% | 1.26% | 2.71% | **87.27%** |
| **73** | Dasos Nanitsa | 1.12% | 30.07% | 25.17% | 1.30% | 42.35% |
| **74** | Dasos Nanitsa | 1.20% | 2.44% | 1.50% | 1.20% | **93.58%** |
| **75** | Dasos Nanitsa | 1.28% | 0.75% | 0.56% | 0.80% | **96.60%** |
| **76** | Dasos Nanitsa | 0.60% | 0.70% | 0.62% | 0.50% | **97.50%** |
| **77** | Dasos Nanitsa | 6.55% | 3.61% | 43.71% | 3.11% | 43.00% |
| **78** | Dasos Nanitsa | 0.60% | 0.70% | 0.70% | 0.90% | **97.16%** |
| **79** | Dasos Nanitsa | 2.32% | 1.00% | 1.78% | 4.01% | **90.92%** |
| **80** | Dasos Nanitsa | 0.90% | 0.91% | 1.00% | 0.70% | **96.50%** |
| **81** | Dasos Nanitsa | 0.69% | 0.60% | 0.50% | 0.50% | **97.70%** |
| **82** | Dasos Nanitsa | 0.60% | 1.85% | 0.40% | 0.80% | **96.45%** |
| **83** | Dasos Nanitsa | 0.60% | 1.27% | 0.60% | 0.50% | **97.09%** |
| **84** | Dasos Nanitsa | 1.90% | 9.09% | 0.80% | 0.63% | **87.52%** |
| **85** | Dasos Nanitsa | 1.03% | 2.46% | 0.68% | 0.56% | **95.28%** |
| **86** | Dasos Nanitsa | 0.50% | 0.60% | 0.50% | 1.30% | **97.20%** |
| **87** | Dasos Nanitsa | 0.60% | 0.80% | 0.50% | 1.00% | **97.09%** |
| **88** | Dasos Nanitsa | 1.00% | 2.82% | 0.58% | 1.92% | **93.69%** |
| **89** | Dasos Nanitsa | 0.60% | 2.54% | 3.55% | 1.10% | **92.22%** |
| **90** | Dasos Nanitsa | 33.00% | 2.00% | 12.06% | 0.80% | **52.19%** |
| **91** | Dasos Nanitsa | 2.01% | 0.90% | 0.50% | 12.88% | **83.67%** |
| **92** | Dasos Nanitsa | 0.80% | 9.45% | 1.97% | 1.12% | **86.64%** |
| **93** | Dasos Nanitsa | 4.97% | 2.72% | 1.19% | 2.06% | **89.08%** |
| **94** | Dasos Nanitsa | 0.60% | 1.00% | 0.94% | 1.40% | **96.09%** |
| **95** | Dasos Nanitsa | 3.76% | 1.10% | 0.90% | 0.90% | **93.33%** |
| **96** | Dasos Nanitsa | 0.70% | 0.70% | 0.50% | 0.80% | **97.28%** |
| **97** | Dasos Nanitsa | 1.80% | 2.51% | 0.70% | 2.17% | **92.83%** |
| **98** | Dasos Nanitsa | 1.30% | 1.86% | 1.40% | 2.11% | **93.38%** |
| **99** | Dasos Nanitsa | 0.70% | 1.10% | 0.50% | 0.89% | **96.80%** |
| **100** | Dasos Nanitsa | 6.72% | 1.09% | 0.60% | 4.61% | **87.02%** |
| **101** | Dasos Nanitsa | 1.51% | 2.27% | 0.50% | 0.60% | **95.05%** |
| **102** | Dasos Nanitsa | 0.60% | 5.47% | 0.60% | 14.91% | **78.38%** |
| **103** | Dasos Nanitsa | 1.65% | 0.70% | 0.60% | 3.41% | **93.60%** |
| **104** | Dasos Nanitsa | 0.84% | 0.91% | 0.60% | 0.81% | **96.80%** |
| **105** | Dasos Nanitsa | 1.00% | 1.31% | 1.21% | 0.60% | **95.89%** |
| **106** | Dasos Nanitsa | 0.46% | 1.00% | 0.50% | 0.70% | **97.37%** |
| **107** | Dasos Nanitsa | 1.50% | 2.26% | 0.50% | 0.60% | **95.05%** |
| **108** | Dasos Nanitsa | 1.20% | 0.60% | 0.40% | 1.10% | **96.70%** |
| **109** | Dasos Nanitsa | 5.85% | 5.83% | 0.60% | 1.03% | **86.64%** |
| **110** | Dasos Nanitsa | 2.39% | 19.52% | 3.97% | 6.01% | **68.11%** |
| **111** | Dasos Nanitsa | 1.68% | 0.71% | 0.50% | 0.60% | **96.50%** |
| **112** | Dasos Nanitsa | 0.54% | 1.50% | 8.73% | 0.40% | **88.82%** |
| **113** | Dasos Nanitsa | 0.80% | 0.60% | 0.50% | 0.50% | **97.60%** |
| **114** | Dasos Nanitsa | 0.50% | 0.60% | 0.40% | 0.71% | **97.80%** |
| **115** | Dasos Nanitsa | 0.86% | 0.81% | 1.55% | 0.91% | **95.81%** |
| **116** | Dasos Nanitsa | 0.70% | 1.73% | 0.80% | 4.92% | **91.86%** |
| **117** | Dasos Nanitsa | 0.93% | 1.10% | 0.50% | 1.94% | **95.52%** |
| **118** | Dasos Nanitsa | 0.50% | 5.62% | 3.89% | 0.91% | **89.01%** |
| **119** | Dasos Nanitsa | 4.31% | 11.45% | 2.30% | 1.60% | **80.35%** |
| **120** | Dasos Nanitsa | 15.80% | 19.66% | 0.40% | 4.97% | **59.13%** |
| **121** | Dasos Nanitsa | 0.70% | 0.60% | 0.50% | 1.00% | **97.30%** |
| **122** | Dasos Nanitsa | 0.50% | 0.80% | 0.66% | 1.08% | **96.99%** |
| **123** | Dasos Nanitsa | 1.10% | 4.07% | 0.80% | 1.01% | **93.05%** |
| **124** | Dasos Nanitsa | 0.80% | 15.31% | 0.90% | 0.82% | **82.20%** |
| **125** | Dasos Nanitsa | 1.40% | 0.99% | 0.60% | 7.53% | **89.49%** |
| **126** | Dasos Nanitsa | 0.96% | 1.07% | 0.60% | 0.80% | **96.54%** |
| **127** | Dasos Nanitsa | 1.20% | 2.30% | 0.60% | 0.60% | **95.38%** |
| **128** | Dasos Nanitsa | 2.51% | 0.80% | 0.91% | 1.20% | **94.55%** |
| **129** | Dasos Nanitsa | 0.70% | 3.28% | 0.90% | 0.50% | **94.65%** |
| **130** | Dasos Nanitsa | 1.55% | 3.95% | 0.44% | 0.50% | **93.57%** |
| **131** | Dasos Nanitsa | 2.28% | 1.50% | 10.52% | 3.12% | **82.56%** |
| **132** | Dasos Nanitsa | 0.80% | 1.20% | 0.50% | 0.70% | **96.83%** |
| **133** | Dasos Nanitsa | 0.73% | 4.04% | 0.60% | 1.00% | **93.56%** |
| **134** | Dasos Nanitsa | 3.00% | 2.64% | 1.90% | 6.28% | **86.16%** |
| **135** | Dasos Nanitsa | 0.50% | 0.70% | 0.60% | 1.11% | **97.10%** |
| **136** | Dasos Nanitsa | 1.40% | 2.45% | 0.80% | 0.80% | **94.56%** |
| **137** | Dasos Nanitsa | 5.13% | 3.59% | 0.69% | 1.53% | **89.08%** |
| **138** | Dasos Nanitsa | 1.10% | 0.80% | 0.60% | 0.90% | **96.60%** |
| **139** | Dasos Nanitsa | 0.90% | 1.58% | 0.60% | 1.15% | **95.82%** |
| **140** | Dasos Nanitsa | 1.69% | 1.99% | 0.69% | 4.10% | **91.60%** |
| **141** | Dasos Nanitsa | 0.70% | 0.62% | 0.50% | 0.80% | **97.40%** |
| **142** | Dasos Nanitsa | 0.70% | 1.00% | 0.60% | 0.90% | **96.90%** |
| **143** | Dasos Nanitsa | 0.70% | 1.19% | 0.60% | 1.10% | **96.41%** |
| **144** | Dasos Nanitsa | 0.50% | 7.89% | 0.50% | 0.70% | **90.43%** |
| **145** | Dasos Nanitsa | 0.90% | 1.99% | 0.50% | 0.70% | **95.95%** |
| **146** | Dasos Nanitsa | 1.00% | 3.67% | 2.48% | 1.10% | **91.79%** |
| **147** | Dasos Nanitsa | 3.95% | 0.90% | 1.50% | 3.70% | **89.96%** |
| **148** | Dasos Nanitsa | 0.80% | 0.80% | 0.50% | 0.50% | **97.40%** |
| **149** | Dasos Nanitsa | 0.50% | 0.60% | 0.45% | 0.50% | **97.90%** |
| **150** | Dasos Nanitsa | 0.60% | 0.90% | 0.50% | 0.50% | **97.50%** |
| **151** | Dasos Nanitsa | 4.03% | 0.70% | 1.00% | 8.20% | **86.01%** |
| **152** | Dasos Nanitsa | 1.50% | 8.41% | 0.70% | 0.93% | **88.47%** |
| **153** | Dasos Nanitsa | 0.75% | 3.20% | 0.90% | 0.60% | **94.50%** |
| **154** | Dasos Nanitsa | 0.90% | 0.60% | 0.50% | 0.60% | **97.46%** |
| **155** | Vaeni | 67.28% | 1.20% | 3.38% | 5.46% | **22.63%** |
| **156** | Vaeni | 0.69% | 2.27% | 2.74% | 0.98% | **93.36%** |
| **157** | Vaeni | 2.27% | **1.40%** | 1.10% | 1.20% | **94.05%** |
| **158** | Vaeni | 3.07% | **85.79%** | 3.11% | 7.17% | 0.80% |
| **159** | Vaeni | 4.07% | **74.82%** | 5.43% | 5.85% | 9.83% |
| **160** | Vaeni | 2.73% | **93.34%** | 0.70% | 1.29% | 2.00% |
| **161** | Vaeni | **83.79%** | 4.99% | 3.40% | 6.82% | 1.00% |
| **162** | Vaeni | 2.49% | 38.03% | 4.04% | 42.65% | 12.82% |
| **163** | Vaeni | 1.31% | 3.52% | 30.72% | **50.23%** | 14.23% |
| **164** | Vaeni | 10.65% | 25.09% | 0.90% | 2.80% | **60.51%** |
| **165** | Vaeni | 1.80% | 2.39% | 0.90% | 0.80% | **94.09%** |
| **166** | Vaeni | 5.16% | 2.30% | 2.46% | 1.78% | **88.35%** |
| **167** | Vaeni | **81.97%** | 2.10% | 0.97% | 6.38% | 8.58% |
| **168** | Vaeni | 0.90% | 1.10% | 0.50% | 0.50% | **96.91%** |
| **169** | Vaeni | 0.80% | 1.22% | 0.50% | 0.50% | **96.98%** |
| **170** | Vaeni | 0.85% | 0.91% | 0.61% | 0.90% | **96.70%** |
| **171** | Vaeni | 1.30% | 5.65% | 0.60% | 0.70% | **91.75%** |
| **172** | Vaeni | 0.70% | **66.90%** | 2.34% | 2.95% | 27.12% |
| **173** | Vaeni | 1.60% | **76.60%** | 5.19% | 1.08% | 15.51% |
| **174** | Vaeni | 2.53% | **94.45%** | 0.94% | 0.60% | 1.48% |
| **175** | Vaeni | **53.85%** | 43.53% | 0.96% | 0.70% | 0.95% |
| **176** | Vaeni | 1.84% | 38.16% | 33.69% | 2.50% | 23.80% |
| **177** | Vaeni | 15.30% | 15.86% | **65.75%** | 1.00% | 2.13% |
| **178** | Vaeni | 48.69% | 12.57% | 4.62% | 17.90% | 16.19% |
| **179** | Vaeni | 0.80% | 39.40% | **52.41%** | 1.50% | 5.90% |
| **180** | Vaeni | 2.95% | **91.01%** | 1.28% | 0.70% | 4.04% |
| **181** | Vaeni | **57.83%** | 30.79% | 1.40% | 3.86% | 6.12% |
| **182** | Vaeni | **67.14%** | 10.26% | 21.18% | 0.70% | 0.70% |
| **183** | Vaeni | 14.38% | 7.76% | **54.14%** | 2.76% | 20.97% |
| **184** | Mariolata | 1.00% | 3.00% | **90.03%** | 5.22% | 0.70% |
| **185** | Mariolata | 1.40% | 20.77% | **75.58%** | 0.72% | 1.50% |
| **186** | Mariolata | 2.55% | 2.03% | **62.38%** | 15.61% | 17.47% |
| **187** | Mariolata | 1.00% | 0.79% | **97.00%** | 0.54% | 0.70% |
| **188** | Mariolata | 0.51% | 0.41% | **98.00%** | 0.50% | 0.50% |
| **189** | Mariolata | 0.50% | 0.60% | **97.70%** | 0.51% | 0.60% |
| **190** | Mariolata | 1.90% | 0.52% | **94.79%** | 0.50% | 2.28% |
| **191** | Mariolata | 0.40% | 0.50% | **98.19%** | 0.50% | 0.50% |
| **192** | Mariolata | 0.40% | 0.70% | **97.17%** | 0.94% | 0.70% |
| **193** | Mariolata | 7.75% | 1.81% | **85.17%** | 4.44% | 0.80% |
| **194** | Mariolata | 1.48% | 2.58% | 45.53% | 44.21% | 6.19% |
| **195** | Mariolata | 0.40% | 1.19% | **97.59%** | 0.40% | 0.50% |
| **196** | Mariolata | 0.60% | 1.00% | **97.50%** | 0.50% | 0.40% |
| **197** | Mariolata | 0.40% | 0.70% | **95.46%** | 0.92% | 2.51% |
| **198** | Mariolata | 1.11% | 0.92% | **85.80%** | 11.57% | 0.60% |
| **199** | Mariolata | 0.50% | 0.60% | **98.10%** | 0.40% | 0.40% |
| **200** | Mariolata | 6.18% | 1.42% | **89.22%** | 1.99% | 1.20% |
| **201** | Mariolata | 0.50% | 0.50% | **98.14%** | 0.50% | 0.40% |
| **202** | Mariolata | 0.40% | 0.49% | **98.10%** | 0.59% | 0.46% |
| **203** | Mariolata | 0.50% | 0.50% | **98.18%** | 0.40% | 0.40% |
| **204** | Mariolata | 1.00% | 1.07% | **96.73%** | 0.61% | 0.55% |
| **205** | Mariolata | 0.60% | 0.50% | **97.78%** | 0.50% | 0.60% |
| **206** | Mariolata | 1.58% | 1.25% | **63.39%** | 26.51% | 7.32% |
| **207** | Mariolata | 7.21% | 3.68% | **69.54%** | 3.76% | 15.82% |
| **208** | Mariolata | 3.49% | 2.61% | 47.57% | 11.89% | 34.46% |
| **209** | Mariolata | 1.49% | 1.97% | **78.18%** | 6.84% | 11.53% |
| **210** | Mariolata | 0.90% | 1.10% | **91.48%** | 1.19% | 5.37% |
| **211** | Mariolata | 0.60% | 0.70% | **97.50%** | 0.50% | 0.70% |
| **212** | Mariolata | 0.53% | 0.69% | **97.80%** | 0.45% | 0.60% |
| **213** | Mariolata | 0.50% | 0.50% | **98.00%** | 0.50% | 0.59% |
| **214** | Mariolata | 0.80% | 0.60% | **97.40%** | 0.60% | 0.70% |
| **215** | Mariolata | 5.26% | 5.28% | **57.36%** | 28.85% | 3.27% |
| **216** | Mariolata | 1.39% | 0.60% | **96.60%** | 0.56% | 0.90% |
| **217** | Mariolata | 1.00% | 0.60% | **97.10%** | 0.50% | 0.80% |
| **218** | Mariolata | 1.23% | 1.00% | **95.60%** | 0.71% | 1.40% |
| **219** | Mariolata | 0.50% | 0.40% | **98.10%** | 0.51% | 0.50% |
| **220** | Mariolata | 0.40% | 0.50% | **98.21%** | 0.46% | 0.50% |
| **221** | Mariolata | 0.40% | 0.50% | **98.17%** | 0.48% | 0.50% |
| **222** | Mariolata | 2.07% | 0.44% | **68.82%** | 26.62% | 2.02% |
| **223** | Mariolata | 1.09% | 0.80% | **97.30%** | 0.40% | 0.40% |
| **224** | Mariolata | 0.50% | 0.80% | **97.77%** | 0.50% | 0.50% |
| **225** | Mariolata | 0.80% | 1.48% | **95.46%** | 0.51% | 1.70% |
| **226** | Mariolata | 2.17% | 1.20% | **76.54%** | 1.26% | 18.82% |
| **227** | Mariolata | 0.78% | 0.40% | **97.60%** | 0.60% | 0.60% |
| **228** | Mariolata | 2.45% | 4.63% | **90.10%** | 1.00% | 1.81% |
| **229** | Mariolata | 0.90% | 0.50% | **97.42%** | 0.50% | 0.70% |
| **230** | Karitsa_I | 2.47% | 2.20% | 1.05% | **92.01%** | 2.29% |
| **231** | Karitsa_I | 6.96% | 12.65% | 35.67% | 30.29% | 14.41% |
| **232** | Karitsa_I | 0.42% | 0.60% | 1.00% | **92.76%** | 5.23% |
| **233** | Karitsa_I | 0.60% | 0.80% | 0.70% | **97.32%** | 0.68% |
| **234** | Karitsa_I | 1.42% | 0.71% | 0.82% | **92.07%** | 4.93% |
| **235** | Karitsa_I | 0.80% | 2.11% | 0.60% | **94.86%** | 1.60% |
| **236** | Karitsa_I | 0.80% | 0.90% | 1.00% | **96.10%** | 1.20% |
| **237** | Karitsa_I | 1.69% | 0.60% | 1.30% | **94.86%** | 1.60% |
| **238** | Karitsa_I | 1.20% | 0.60% | 1.66% | **94.90%** | 1.62% |
| **239** | Karitsa_I | 0.50% | 0.70% | 0.50% | **97.67%** | 0.70% |
| **240** | Karitsa_I | 2.30% | 0.60% | 0.90% | **95.45%** | 0.80% |
| **241** | Karitsa_I | 0.59% | 3.20% | 1.50% | **89.70%** | 5.00% |
| **242** | Karitsa_I | 1.35% | 1.58% | 0.80% | 2.25% | **94.00%** |
| **243** | Karitsa_I | 0.80% | 1.63% | 1.00% | 18.70% | **77.88%** |
| **244** | Karitsa_I | 1.40% | 2.73% | 0.70% | 5.91% | **89.28%** |
| **245** | Karitsa_I | 37.33% | 9.37% | 1.32% | 1.62% | **50.33%** |
| **246** | Karitsa_I | 7.32% | 45.41% | 0.60% | 2.64% | 43.98% |
| **247** | Karitsa_I | 1.93% | 1.81% | 1.10% | 1.20% | **93.97%** |
| **248** | Karitsa_I | 6.38% | 11.60% | 38.23% | 2.88% | 40.88% |
| **249** | Karitsa_I | 7.48% | 3.07% | 0.60% | 38.60% | **50.28%** |
| **250** | Karitsa_I | 1.76% | 3.84% | 0.60% | 37.94% | **55.83%** |
| **251** | Karitsa_I | 1.50% | 48.47% | 0.90% | 43.23% | 5.96% |
| **252** | Karitsa_I | 0.81% | 6.33% | 2.34% | 23.02% | **67.47%** |
| **253** | Karitsa_I | 2.60% | 1.30% | 0.90% | **94.44%** | 0.80% |
| **254** | Karitsa_I | 3.91% | 1.91% | 0.50% | **90.94%** | 2.73% |
| **255** | Karitsa_I | 0.80% | 0.50% | 0.50% | **97.60%** | 0.50% |
| **256** | Karitsa_I | 2.30% | 0.70% | 0.60% | **74.43%** | 22.00% |
| **257** | Karitsa_I | 0.70% | 0.40% | 0.50% | **97.80%** | 0.60% |
| **258** | Karitsa_I | 1.05% | 1.80% | 14.16% | **82.17%** | 0.80% |
| **259** | Karitsa_I | 0.60% | 0.54% | 0.80% | **97.60%** | 0.50% |
| **260** | Karitsa_I | 0.50% | 0.50% | 0.40% | **98.00%** | 0.50% |
| **261** | Karitsa_I | 0.60% | 0.60% | 0.40% | **97.70%** | 0.70% |
| **262** | Karitsa_I | 0.50% | 0.53% | 0.40% | **97.98%** | 0.50% |
| **263** | Karitsa_I | 1.01% | 26.45% | 0.50% | **68.72%** | 3.36% |
| **264** | Karitsa_I | 0.57% | 0.60% | 0.40% | **97.99%** | 0.50% |
| **265** | Karitsa_I | 1.09% | 1.00% | 1.10% | **94.27%** | 2.53% |
| **266** | Karitsa_I | 0.70% | 1.10% | 0.68% | **95.81%** | 1.70% |
| **267** | Karitsa_I | 0.60% | 0.70% | 8.35% | **88.28%** | 2.06% |
| **268** | Karitsa_I | 0.51% | 2.69% | 0.60% | **95.28%** | 0.90% |
| **269** | Karitsa_I | 1.24% | 0.70% | 1.04% | **94.82%** | 2.19% |
| **270** | Karitsa_I | 1.10% | 1.70% | 1.77% | **88.31%** | 7.15% |
| **271** | Karitsa_I | 0.60% | 2.31% | 0.60% | **89.23%** | 7.29% |
| **272** | Karitsa_I | 3.68% | 0.70% | 0.60% | **94.04%** | 1.00% |
| **273** | Karitsa_I | 5.80% | 5.31% | 0.60% | 27.91% | **60.44%** |
| **274** | Karitsa_I | 0.60% | 4.07% | 4.44% | 1.58% | **89.29%** |
| **275** | Karitsa_I | 1.21% | 7.72% | 0.60% | **88.85%** | 1.61% |
| **276** | Karitsa_I | 0.79% | 1.49% | 4.77% | **85.71%** | 7.25% |
| **277** | Karitsa_I | 0.70% | 0.60% | 0.40% | **97.80%** | 0.50% |
| **278** | Karitsa_I | 0.40% | 0.60% | 0.60% | **97.80%** | 0.60% |
| **279** | Karitsa_I | 0.62% | 0.50% | 0.50% | **97.81%** | 0.60% |
| **280** | Karitsa_I | 0.61% | 0.50% | 0.60% | **97.40%** | 0.80% |
| **281** | Karitsa_I | 0.80% | 1.10% | 0.40% | **97.09%** | 0.61% |
| **282** | Karitsa_I | 0.70% | 0.70% | 0.50% | **97.10%** | 0.91% |
| **283** | Karitsa_I | 2.29% | 3.57% | 0.60% | 7.21% | **86.30%** |
| **284** | Karitsa_I | 0.60% | 0.50% | 1.00% | **97.30%** | 0.60% |
| **285** | Karitsa_I | 0.90% | 0.70% | 0.50% | **97.25%** | 0.70% |
| **286** | Karitsa_I | 0.50% | 0.80% | 7.74% | **88.76%** | 2.21% |
| **287** | Karitsa_I | 10.89% | 3.62% | 3.85% | **69.71%** | 11.91% |
| **288** | Karitsa_I | 1.60% | 1.57% | 0.70% | **95.02%** | 1.10% |
| **289** | Karitsa_I | 0.60% | 0.73% | 0.40% | **97.60%** | 0.60% |
| **290** | Karitsa_I | 1.64% | 0.71% | 4.06% | **92.44%** | 1.10% |
| **291** | Karitsa_I | 1.17% | 2.10% | 2.48% | **90.78%** | 3.50% |
| **292** | Karitsa_I | 1.38% | 1.29% | 7.96% | **88.01%** | 1.40% |
| **293** | Karitsa_I | 1.00% | 1.02% | 0.50% | **96.51%** | 1.00% |
| **294** | Karitsa_I | 0.60% | 2.00% | 0.50% | **95.93%** | 1.00% |
| **295** | Karitsa_I | 1.20% | 0.50% | 1.10% | **96.63%** | 0.60% |
| **296** | Karitsa_I | 1.49% | 0.60% | 0.78% | **96.64%** | 0.50% |
| **297** | Karitsa_I | 2.09% | 1.80% | 0.55% | **94.32%** | 1.21% |
| **298** | Karitsa_I | 6.11% | 4.69% | 0.70% | **87.73%** | 0.80% |
| **299** | Karitsa_I | 1.77% | 1.55% | 34.92% | **54.09%** | 7.66% |
| **300** | Karitsa_I | 0.50% | 0.70% | 0.80% | **97.40%** | 0.60% |
| **301** | Karitsa_I | 0.80% | 0.50% | 0.50% | **97.59%** | 0.70% |
| **302** | Karitsa_I | 1.02% | 0.70% | 0.43% | **97.20%** | 0.70% |
| **303** | Karitsa_I | 0.60% | 0.60% | 0.40% | **97.80%** | 0.60% |
| **304** | Karitsa_I | 0.60% | 0.50% | 0.50% | **97.90%** | 0.50% |
| **305** | Karitsa_I | 0.50% | 0.60% | 0.40% | **97.70%** | 0.80% |
| **306** | Karitsa_I | 0.63% | 0.40% | 0.50% | **97.80%** | 0.60% |
| **307** | Karitsa_I | 2.29% | 6.17% | 8.07% | **79.85%** | 3.58% |
| **308** | Karitsa_I | 0.70% | 1.30% | 0.51% | **96.77%** | 0.80% |
| **309** | Karitsa_I | 1.20% | 2.37% | 2.94% | **90.67%** | 2.81% |
| **310** | Karitsa_I | 0.50% | 0.50% | 0.50% | **97.80%** | 0.61% |
| **311** | Karitsa_I | 1.76% | 0.58% | 0.50% | **96.29%** | 0.96% |
| **312** | Karitsa_I | 1.55% | 14.68% | 3.24% | **61.54%** | 18.99% |
| **313** | Karitsa_I | 0.50% | 0.60% | 0.55% | **97.70%** | 0.70% |
| **314** | Karitsa_I | 0.80% | 0.60% | 0.50% | **97.40%** | 0.78% |
| **315** | Karitsa_I | 0.80% | 0.60% | 0.80% | **95.92%** | 1.87% |
| **316** | Karitsa_I | 1.72% | 1.70% | 1.20% | **91.59%** | 3.75% |
| **317** | Karitsa_I | 1.44% | 2.35% | 0.80% | **79.39%** | 16.07% |
| **318** | Karitsa_I | 1.20% | 0.70% | 1.03% | **96.39%** | 0.70% |
| **319** | Karitsa_I | 0.92% | 2.16% | 1.60% | **90.68%** | 4.62% |
| **320** | Karitsa_I | 1.81% | 0.60% | 0.80% | **96.10%** | 0.70% |
| **321** | Karitsa_I | 4.40% | 0.70% | 0.70% | **68.69%** | 25.50% |
| **322** | Karitsa_I | 0.90% | 1.23% | 0.57% | **96.67%** | 0.61% |
| **323** | Karitsa_I | 0.80% | 1.00% | 0.41% | **97.17%** | 0.60% |
| **324** | Karitsa_II | 0.75% | 1.95% | 1.90% | **92.18%** | 3.25% |
| **325** | Karitsa_II | 0.71% | 0.60% | 0.50% | **97.40%** | 0.80% |
| **326** | Karitsa_II | 1.22% | 0.50% | 0.68% | **96.71%** | 0.88% |
| **327** | Karitsa_II | 0.80% | 0.63% | 0.70% | **97.22%** | 0.60% |
| **328** | Karitsa_II | 1.40% | 0.71% | 0.63% | **96.31%** | 0.90% |
| **329** | Karitsa_II | 3.27% | 1.88% | 0.70% | **91.60%** | 2.56% |
| **330** | Karitsa_II | 1.03% | 0.70% | 1.13% | **96.48%** | 0.70% |
| **331** | Karitsa_II | 0.60% | 0.77% | 0.50% | **95.63%** | 2.53% |
| **332** | Karitsa_II | 0.80% | 0.70% | 0.50% | **97.40%** | 0.60% |
| **333** | Karitsa_II | 0.50% | 0.82% | 0.40% | **97.67%** | 0.60% |
| **334** | Karitsa_II | 0.89% | 0.83% | 0.50% | **94.41%** | 3.42% |
| **335** | Karitsa_II | 0.50% | 0.50% | 0.50% | **97.81%** | 0.60% |
| **336** | Karitsa_II | 6.78% | 28.17% | 0.79% | **53.62%** | 10.66% |
| **337** | Karitsa_II | 0.69% | 1.20% | 0.70% | **96.70%** | 0.70% |
| **338** | Karitsa_II | 2.86% | 0.80% | 1.10% | **94.38%** | 0.82% |
| **339** | Karitsa_II | 2.22% | 1.40% | 0.60% | **94.99%** | 0.80% |
| **340** | Karitsa_II | 2.01% | 1.20% | 0.79% | **90.02%** | 5.97% |
| **341** | Karitsa_II | 0.60% | 0.70% | 0.90% | **97.23%** | 0.60% |
| **342** | Karitsa_II | 1.50% | 0.70% | 0.40% | **96.90%** | 0.50% |
| **343** | Karitsa_II | 1.57% | 1.13% | 0.60% | **95.79%** | 0.90% |
| **344** | Karitsa_II | 6.84% | 9.54% | 3.29% | **65.72%** | 14.60% |
| **345** | Karitsa_II | 1.10% | 5.11% | 1.38% | **91.66%** | 0.74% |
| **346** | Vathirrevma | 1.80% | **96.48%** | 0.40% | 0.70% | 0.60% |
| **347** | Vathirrevma | 1.40% | **95.21%** | 1.70% | 0.70% | 1.00% |
| **348** | Vathirrevma | 1.04% | 31.71% | 2.00% | **64.67%** | 0.60% |
| **349** | Vathirrevma | 0.80% | **96.90%** | 0.94% | 0.70% | 0.60% |
| **350** | Vathirrevma | 3.10% | **89.59%** | 1.10% | 4.79% | 1.40% |
| **351** | Vathirrevma | 3.16% | **86.19%** | 1.70% | 7.88% | 1.09% |
| **352** | Vathirrevma | 0.60% | **84.93%** | 3.04% | 1.30% | 10.10% |
| **353** | Vathirrevma | 2.62% | **83.83%** | 0.80% | 11.99% | 0.80% |
| **354** | Vathirrevma | 4.09% | **93.55%** | 0.50% | 0.90% | 1.00% |
| **355** | Vathirrevma | 0.75% | **93.24%** | 0.70% | 0.90% | 4.43% |
| **356** | Vathirrevma | 7.69% | **57.91%** | 2.01% | 23.51% | 8.88% |
| **357** | Vathirrevma | 0.60% | **97.60%** | 0.50% | 0.60% | 0.70% |
| **358** | Vathirrevma | 0.70% | **96.90%** | 0.60% | 0.60% | 1.20% |
| **359** | Vathirrevma | 0.80% | **96.90%** | 0.70% | 0.80% | 0.80% |
| **360** | Vathirrevma | 0.70% | **96.80%** | 0.70% | 0.70% | 1.05% |
| **361** | Vathirrevma | 49.03% | 39.25% | 0.70% | 5.37% | 5.68% |
| **362** | Vathirrevma | 16.72% | **79.16%** | 0.90% | 2.50% | 0.79% |
| **363** | Vathirrevma | 0.80% | **97.18%** | 0.50% | 0.72% | 0.79% |
| **364** | Vathirrevma | 4.90% | **86.81%** | 2.20% | 1.60% | 4.49% |
| **365** | Vathirrevma | 3.98% | **89.39%** | 2.31% | 1.31% | 3.00% |
| **366** | Vathirrevma | 1.16% | **94.34%** | 1.20% | 1.88% | 1.40% |
| **367** | Vathirrevma | 1.00% | **56.09%** | 38.93% | 1.00% | 3.01% |
| **368** | Vathirrevma | 0.60% | **97.30%** | 0.50% | 0.50% | 1.10% |
| **369** | Vathirrevma | 0.60% | **95.07%** | 2.92% | 0.70% | 0.70% |
| **370** | Vathirrevma | 8.30% | **73.05%** | 11.16% | 0.94% | 6.52% |
| **371** | Vathirrevma | 0.90% | **95.62%** | 1.00% | 0.70% | 1.70% |
| **372** | Vathirrevma | 5.97% | 18.97% | **52.95%** | 1.21% | 20.86% |
| **373** | Vathirrevma | 1.51% | **84.28%** | 3.21% | 4.01% | 6.97% |
| **374** | Vathirrevma | 1.69% | **93.78%** | 0.60% | 1.12% | 2.79% |
| **375** | Vathirrevma | 1.32% | **96.40%** | 0.70% | 1.00% | 0.60% |
| **376** | Vathirrevma | 0.70% | **97.00%** | 0.70% | 0.99% | 0.60% |
| **377** | Vathirrevma | 2.34% | **89.40%** | 0.95% | 5.78% | 1.51% |
| **378** | Vathirrevma | 0.90% | **93.33%** | 2.63% | 0.80% | 2.39% |
| **379** | Vathirrevma | 3.13% | **92.85%** | 1.00% | 0.90% | 2.07% |
| **380** | Vathirrevma | 2.20% | **79.22%** | 14.25% | 0.90% | 3.48% |
| **381** | Vathirrevma | 0.60% | **96.87%** | 0.60% | 0.90% | 1.10% |
| **382** | Vathirrevma | 2.96% | **93.06%** | 0.70% | 0.71% | 2.57% |
| **383** | Vathirrevma | 0.40% | **89.85%** | 0.70% | 2.96% | 6.07% |
| **384** | Vathirrevma | 0.80% | **97.00%** | 0.80% | 0.50% | 0.90% |
| **385** | Vathirrevma | 1.50% | **81.65%** | 8.80% | 7.04% | 1.00% |
| **386** | Vathirrevma | 2.67% | **94.77%** | 0.60% | 0.70% | 1.23% |
| **387** | Vathirrevma | 0.60% | **95.50%** | 0.60% | 1.09% | 2.28% |
| **388** | Vathirrevma | 1.36% | **95.47%** | 0.90% | 0.50% | 1.82% |
| **389** | Vathirrevma | 3.17% | **85.06%** | 0.60% | 2.37% | 8.82% |
| **390** | Vathirrevma | 0.70% | **96.90%** | 0.80% | 0.70% | 0.80% |
| **391** | Vathirrevma | 0.50% | **97.40%** | 0.50% | 0.70% | 0.90% |
| **392** | Vathirrevma | 0.40% | **95.98%** | 2.49% | 0.50% | 0.60% |
| **393** | Vathirrevma | 1.51% | **94.39%** | 0.90% | 0.80% | 2.39% |
| **394** | Vathirrevma | 0.76% | **96.05%** | 1.10% | 0.80% | 1.30% |
| **395** | Vathirrevma | 0.60% | **95.39%** | 0.50% | 1.80% | 1.70% |
| **396** | Vathirrevma | 0.50% | **79.86%** | 0.50% | 17.98% | 1.10% |
| **397** | Vathirrevma | 2.52% | **70.29%** | 1.10% | 5.54% | 20.58% |
| **398** | Vathirrevma | 0.50% | **96.94%** | 0.92% | 0.60% | 1.00% |
| **399** | Vathirrevma | 1.10% | **96.04%** | 0.60% | 0.60% | 1.60% |
| **400** | Vathirrevma | 1.00% | **94.64%** | 0.80% | 1.10% | 2.43% |
| **401** | Vathirrevma | 2.86% | **94.90%** | 0.50% | 0.90% | 0.80% |
| **402** | Vathirrevma | 1.36% | **91.48%** | 1.00% | 4.60% | 1.53% |
| **403** | Vathirrevma | 1.21% | **95.46%** | 1.00% | 1.22% | 1.10% |
| **404** | Vathirrevma | 1.40% | **91.69%** | 0.78% | 5.35% | 0.80% |
| **405** | Vathirrevma | 2.28% | **95.51%** | 0.60% | 0.90% | 0.71% |
| **406** | Vathirrevma | 1.36% | **96.19%** | 0.70% | 0.50% | 1.30% |
| **407** | Vathirrevma | 2.38% | **93.33%** | 0.85% | 2.26% | 1.20% |
| **408** | Vathirrevma | 1.98% | **73.52%** | 1.10% | 0.88% | 22.54% |
| **409** | Vathirrevma | 0.58% | **96.00%** | 0.77% | 0.70% | 1.97% |
| **410** | Vathirrevma | 0.77% | **96.80%** | 0.90% | 0.75% | 0.80% |
| **411** | Vathirrevma | 2.69% | **93.98%** | 1.10% | 0.68% | 1.54% |
| **412** | Vathirrevma | 0.80% | **96.70%** | 0.50% | 1.20% | 0.80% |
| **413** | Vathirrevma | 0.82% | **97.20%** | 0.50% | 0.50% | 0.90% |
| **414** | Vathirrevma | 0.60% | **96.80%** | 0.60% | 0.50% | 1.51% |
| **415** | Vathirrevma | 0.60% | **97.39%** | 0.50% | 0.60% | 0.90% |
| **416** | Vathirrevma | 0.80% | **96.69%** | 0.90% | 0.70% | 1.00% |
| **417** | Vathirrevma | 1.11% | **68.72%** | 4.27% | 23.15% | 2.72% |
| **418** | Vathirrevma | 1.20% | **92.13%** | 0.92% | 2.91% | 2.83% |
| **419** | Vathirrevma | 0.70% | **97.29%** | 0.72% | 0.70% | 0.60% |
| **420** | Vathirrevma | 0.60% | **96.80%** | 0.80% | 0.90% | 0.90% |
| **421** | Perivoli | 40.63% | 2.59% | 0.80% | **52.37%** | 3.63% |
| **422** | Perivoli | **96.60%** | 0.60% | 0.69% | 1.15% | 1.00% |
| **423** | Perivoli | **94.09%** | 2.10% | 2.11% | 0.90% | 0.80% |
| **424** | Perivoli | **98.00%** | 0.60% | 0.50% | 0.50% | 0.40% |
| **425** | Perivoli | **58.57%** | 9.16% | 1.31% | 20.79% | 10.12% |
| **426** | Perivoli | **98.10%** | 0.60% | 0.40% | 0.40% | 0.50% |
| **427** | Perivoli | **96.40%** | 1.37% | 0.80% | 0.52% | 0.90% |
| **428** | Perivoli | **93.05%** | 4.01% | 0.61% | 1.49% | 0.86% |
| **429** | Perivoli | **88.91%** | 0.80% | 1.10% | 6.06% | 3.19% |
| **430** | Perivoli | **72.77%** | 1.60% | 0.44% | 24.36% | 0.82% |
| **431** | Perivoli | **82.21%** | 3.74% | 0.58% | 4.80% | 8.67% |
| **432** | Perivoli | **96.69%** | 1.00% | 0.60% | 0.80% | 0.90% |
| **433** | Perivoli | **90.56%** | 4.78% | 0.60% | 0.70% | 3.33% |
| **434** | Perivoli | **97.11%** | 0.52% | 0.70% | 0.89% | 0.70% |
| **435** | Perivoli | **81.80%** | 3.53% | 0.60% | 7.46% | 6.60% |
| **436** | Perivoli | **97.40%** | 0.70% | 0.50% | 0.80% | 0.60% |
| **437** | Perivoli | **97.30%** | 0.60% | 0.40% | 0.82% | 0.80% |
| **438** | Perivoli | **97.70%** | 0.64% | 0.40% | 0.60% | 0.60% |
| **439** | Perivoli | **85.77%** | 4.32% | 6.34% | 0.70% | 2.85% |
| **440** | Perivoli | **92.41%** | 1.10% | 0.50% | 0.70% | 5.28% |
| **441** | Perivoli | **95.96%** | 0.80% | 0.50% | 1.81% | 1.00% |
| **442** | Perivoli | **94.47%** | 2.57% | 0.70% | 1.34% | 0.90% |
| **443** | Perivoli | **87.11%** | 5.21% | 0.40% | 2.09% | 5.20% |
| **444** | Perivoli | **95.75%** | 0.90% | 1.30% | 1.00% | 1.08% |
| **445** | Perivoli | **83.72%** | 3.62% | 4.43% | 1.19% | 7.07% |
| **446** | Perivoli | **91.10%** | 1.70% | 0.40% | 1.91% | 4.90% |
| **447** | Perivoli | **96.15%** | 1.00% | 0.70% | 0.90% | 1.20% |
| **448** | Perivoli | **95.56%** | 1.00% | 1.30% | 0.90% | 1.27% |
| **449** | Perivoli | **97.90%** | 0.50% | 0.50% | 0.52% | 0.58% |
| **450** | Perivoli | **82.31%** | 2.96% | 1.30% | 12.56% | 0.90% |
| **451** | Perivoli | **74.04%** | 1.00% | 0.80% | 23.09% | 1.09% |
| **452** | Perivoli | **73.09%** | 14.32% | 10.50% | 0.50% | 1.60% |
| **453** | Perivoli | 23.32% | **53.88%** | 0.52% | 12.81% | 9.48% |
| **454** | Perivoli | **65.99%** | 2.10% | 29.79% | 1.30% | 0.80% |
| **455** | Perivoli | **88.91%** | 6.12% | 0.50% | 3.77% | 0.70% |
| **456** | Perivoli | **93.35%** | 1.80% | 0.40% | 3.12% | 1.28% |
| **457** | Perivoli | 30.86% | 9.44% | 20.25% | 11.62% | 27.83% |
| **458** | Perivoli | 4.55% | 91.25% | 0.50% | 1.62% | 2.04% |
| **459** | Perivoli | **52.88%** | 40.36% | 1.39% | 1.00% | 4.38% |
| **460** | Perivoli | **97.28%** | 0.70% | 0.60% | 0.70% | 0.80% |
| **461** | Perivoli | **97.59%** | 0.60% | 0.40% | 0.50% | 0.90% |
| **462** | Perivoli | **98.00%** | 0.50% | 0.50% | 0.50% | 0.50% |
| **463** | Perivoli | **98.20%** | 0.50% | 0.41% | 0.40% | 0.50% |
| **464** | Perivoli | **97.80%** | 0.51% | 0.55% | 0.55% | 0.60% |
| **465** | Perivoli | 7.21% | **90.69%** | 0.40% | 0.86% | 0.80% |
| **466** | Perivoli | 1.37% | **95.63%** | 0.70% | 0.65% | 1.62% |
| **467** | Perivoli | **58.04%** | 31.51% | 2.10% | 5.45% | 2.90% |
| **468** | Perivoli | **85.80%** | 6.92% | 0.60% | 0.77% | 5.90% |
| **469** | Perivoli | **91.85%** | 2.88% | 2.09% | 1.30% | 1.90% |
| **470** | Perivoli | **87.69%** | 3.85% | 1.67% | 5.62% | 1.20% |
| **471** | Perivoli | **96.96%** | 0.80% | 0.50% | 1.25% | 0.60% |
| **472** | Perivoli | **96.40%** | 0.80% | 1.10% | 0.70% | 0.95% |
| **473** | Perivoli | **93.35%** | 2.39% | 0.69% | 1.65% | 1.96% |
| **474** | Perivoli | **86.15%** | 7.55% | 0.50% | 2.84% | 2.93% |
| **475** | Perivoli | **96.99%** | 0.50% | 0.40% | 1.36% | 0.70% |
| **476** | Perivoli | **95.00%** | 1.96% | 0.50% | 1.89% | 0.70% |
| **477** | Perivoli | **97.00%** | 0.80% | 0.80% | 0.54% | 0.90% |
| **478** | Perivoli | **97.80%** | 0.60% | 0.50% | 0.50% | 0.60% |
| **479** | Perivoli | **98.10%** | 0.50% | 0.40% | 0.50% | 0.50% |
| **480** | Perivoli | **97.90%** | 0.60% | 0.44% | 0.50% | 0.50% |
| **481** | Perivoli | **97.60%** | 0.75% | 0.50% | 0.60% | 0.60% |
| **482** | Perivoli | **97.60%** | 0.70% | 0.60% | 0.60% | 0.60% |
| **483** | Perivoli | **97.80%** | 0.60% | 0.50% | 0.50% | 0.50% |
| **484** | Perivoli | **96.41%** | 0.90% | 0.70% | 0.80% | 1.17% |
| **485** | Perivoli | **97.10%** | 0.70% | 0.60% | 0.60% | 0.98% |
| **486** | Perivoli | **96.29%** | 1.60% | 0.52% | 0.80% | 0.80% |
| **487** | Perivoli | **91.86%** | 1.20% | 0.60% | 5.21% | 1.10% |
| **488** | Perivoli | **93.26%** | 1.10% | 0.51% | 4.20% | 0.90% |
| **489** | Perivoli | **96.41%** | 0.70% | 0.80% | 1.17% | 0.94% |
| **490** | Perivoli | 49.34% | 2.73% | 2.46% | 17.79% | 27.69% |
| **491** | Perivoli | **91.09%** | 0.60% | 0.90% | 5.16% | 2.29% |
| **492** | Perivoli | **94.26%** | 2.51% | 1.43% | 0.70% | 1.09% |
| **493** | Perivoli | **85.62%** | 2.58% | 0.71% | 10.33% | 0.71% |
| **494** | Perivoli | **97.50%** | 0.50% | 0.79% | 0.60% | 0.60% |
| **495** | Perivoli | **93.71%** | 3.97% | 0.50% | 0.90% | 0.85% |
